# Supplementary material for: Non-coding RNA Expression, Function, and Variation during Drosophila Embryogenesis
Source: Curr Biol. 2018 Nov 19;28(22):3547–3561.e9. doi: 10.1016/j.cub.2018.09.026 (PMC6264527; doi:10.1016/j.cub.2018.09.026)
Supplement: Document S1. Figures S1–S7 and Tables S1, S6, and S7 [file mmc1.pdf]

Current Biology, Volume *28*

Supplemental Information

Non-coding RNA Expression, Function, and Variation during *Drosophila* Embryogenesis

Ignacio E. Schor, Giovanni Bussotti, Matilda Maleš, Mattia Forneris, Rebecca R. Viales, Anton J. Enright, and Eileen E.M. Furlong

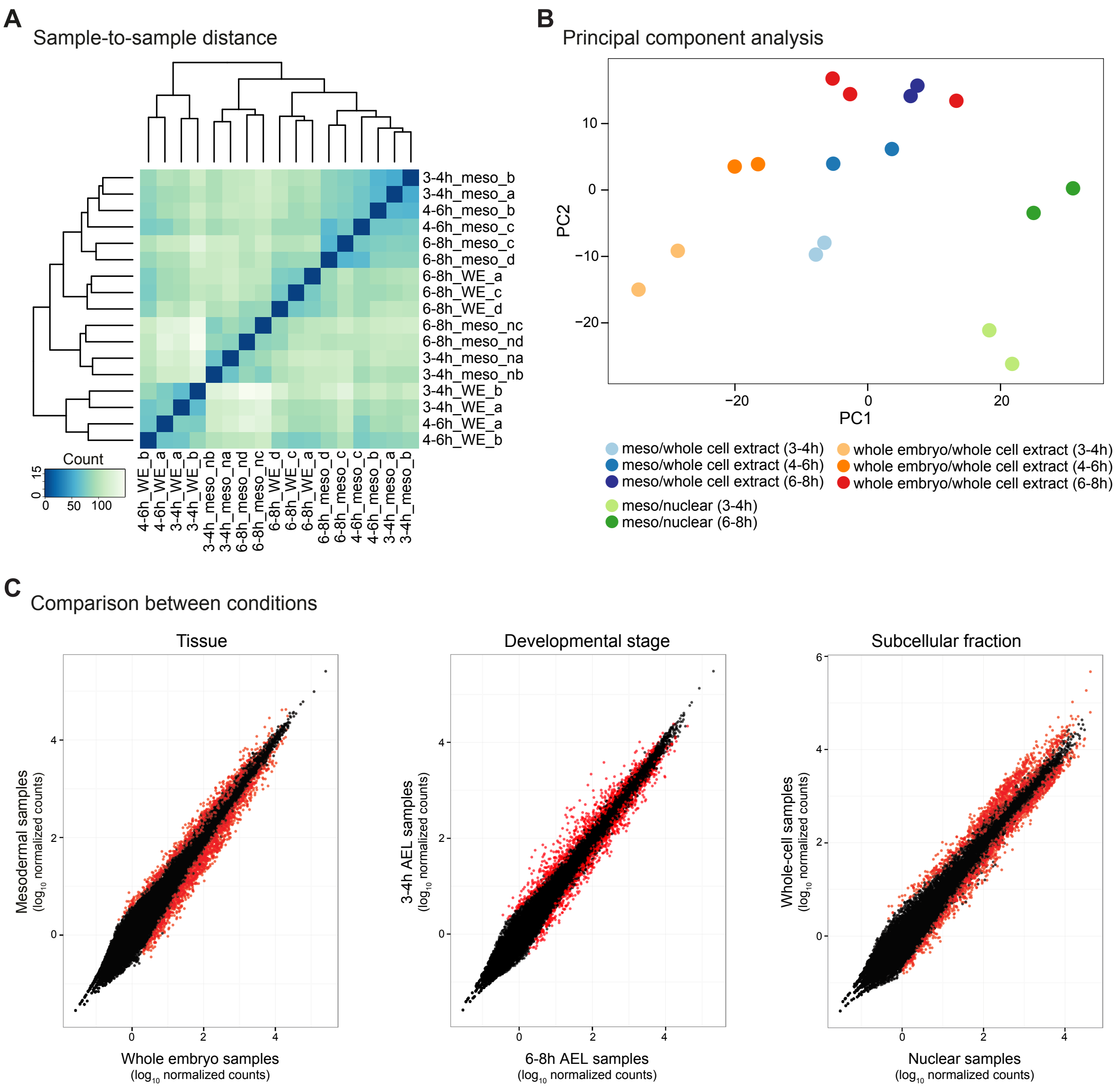

Figure S1. Differential expression analysis. Related to STAR Methods

(A) Matrix of pairwise distances between all samples included in the DE analysis. (B) Principal component analysis. S = FACS sorted (mesoderm), U= unsorted (whole embryo), yes = nuclear enrichment, no = whole cell extract. Replicates group together. Samples tend to group closer by developmental time point, then by tissue or nuclear fraction (e.g. all 3-4hr samples are in the lower half). (C) BaseMean counts (log 10 scale) as estimated by DESeq2 comparing between: *(left)* tissue (mesoderm vs whole embryo), *(middle)* developmental stage, *(right)* nuclear fraction versus whole cell. BaseMean is the mean of normalized counts of all samples of that condition, normalizing for sequencing depth. For example, the middle panel reflects gene coverage at 3-4 vs 6-8 hours, averaging all 3-4h and 6-8h samples (i.e. including both WE and mesoderm samples). The right panel reflects gene coverage in nuclear enriched vs non-nuclear enriched samples, averaging all nuclear and non-nuclear samples (which are all mesoderm samples). Scatterplots show significant genes for the indicated contrasts. Red dots depict genes with adjusted *p*-value < 0.01.

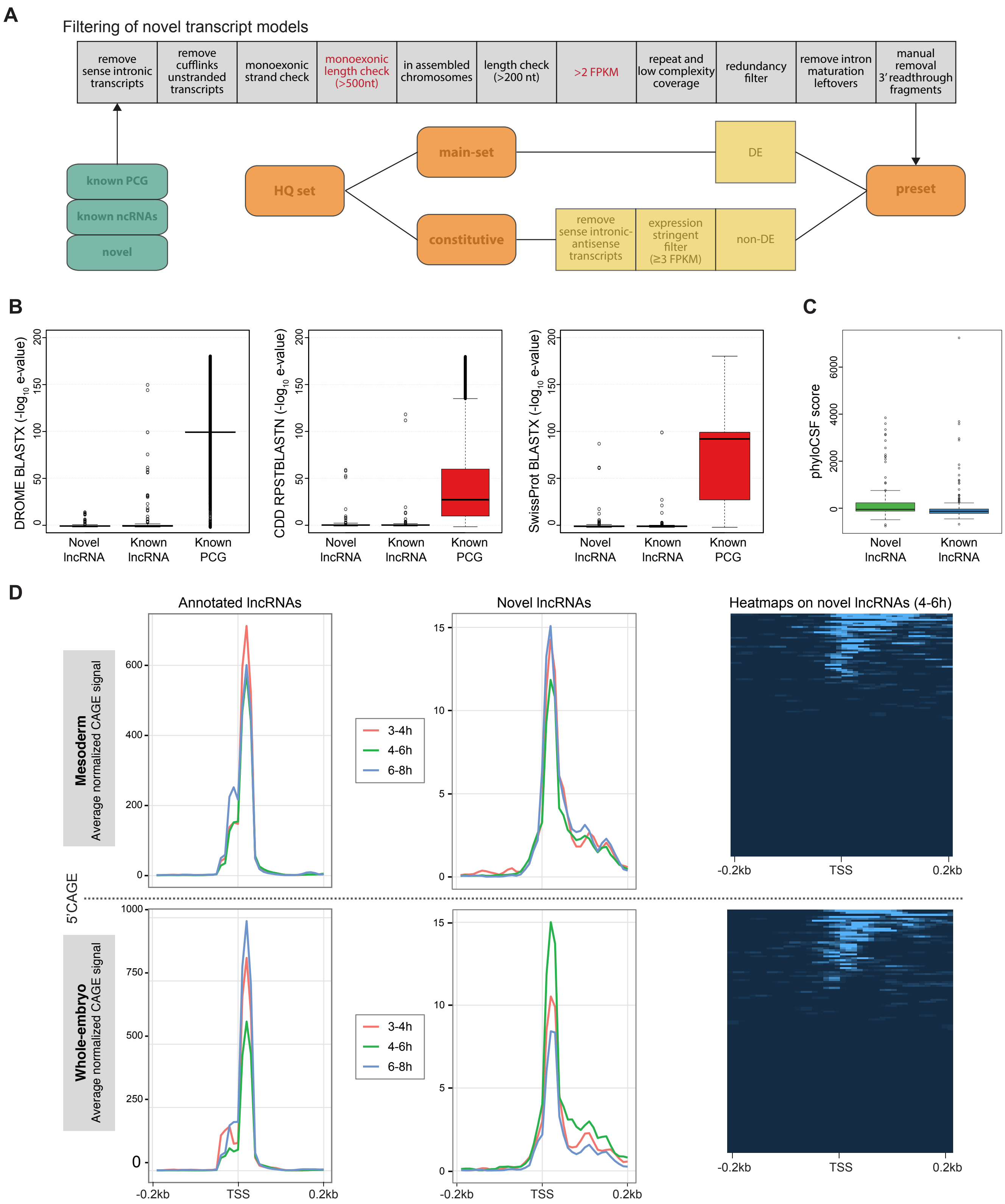

Figure S2. Identification and validation of novel lncRNA genes. Related to Figure 1

(A) Overview of transcript filtering to obtain a high-confidence set of new lncRNAs. The two most stringent filters, in terms of transcripts removed, are shown in red text. The filtered pre-set was divided into transcripts with (main-set) and without (constitutive) significant ($p < 0.01$) differential expression across stage or tissue. The combination of these two results in our high-quality (HQ) set of novel lncRNAs used in the rest of the analysis. (B) Boxplots showing the e-values of the three transcript sets (novel and known lncRNA and PCGs) for the following BLAST searches: BLASTX against Drosophila proteome (DROME) and SwissProt; RPST BLASTN against the NCBI Conserved Domain Database (CDD). (C) PhyloCSF analysis for conservation of predicted ORFs found in the novel and annotated lncRNA sets. (D) 5'CAGE support of transcript start sites. Plots show average 5'CAGE signal for promoter regions of novel and annotated lncRNAs. Transcripts with promoter region overlapping other TSSs (in a window of 50nt on the same strand) were excluded. Heatmaps represent normalized CAGE signal for novel transcripts expressed at 4-6h in mesoderm and whole embryo.

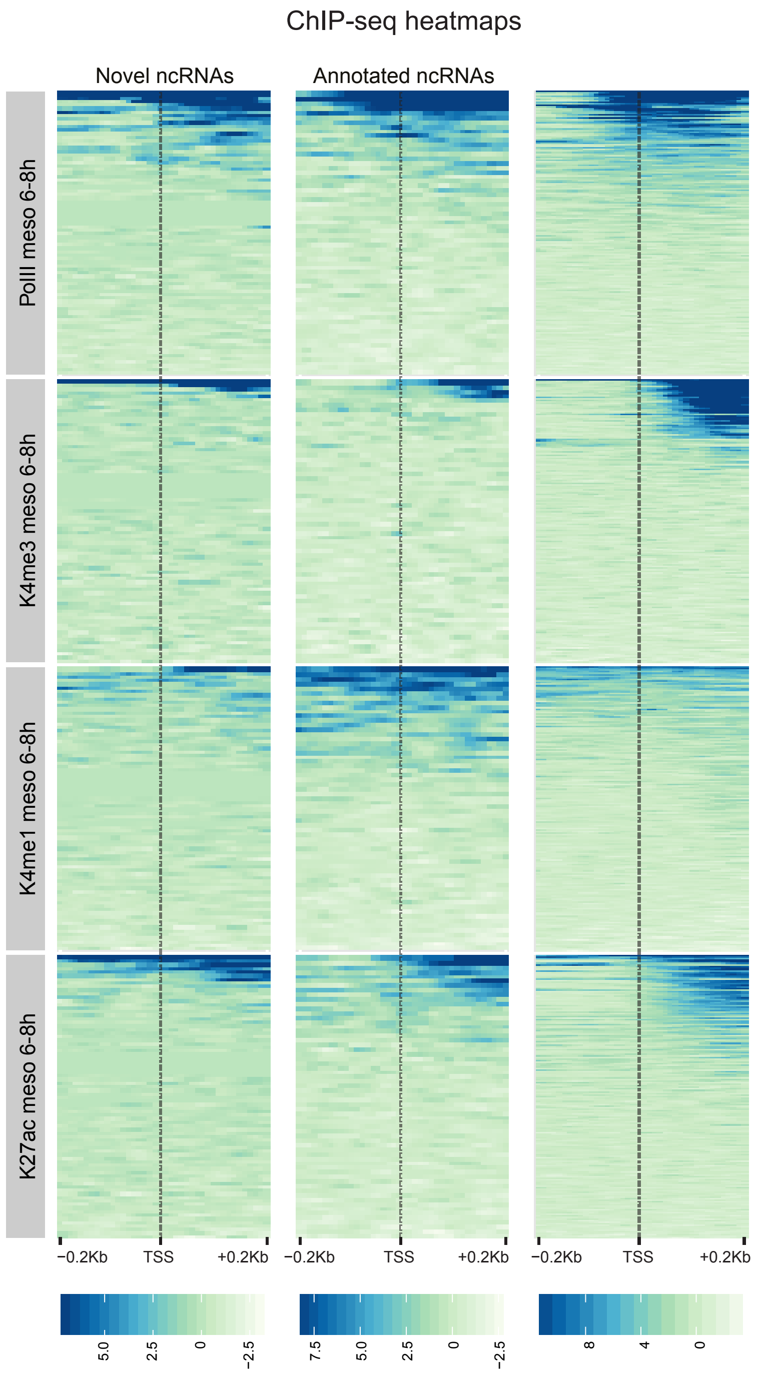

Figure S3. RNA pol II and promoter associated chromatin marks are often not detected at lncRNA promoters. Related to Figure 1

Shown are heatmaps of ChIP-seq signal for the indicated factors at novel lncRNA, annotated lncRNA and protein-coding gene promoters. Data corresponds to the average profile plots shown in Figure 1D.

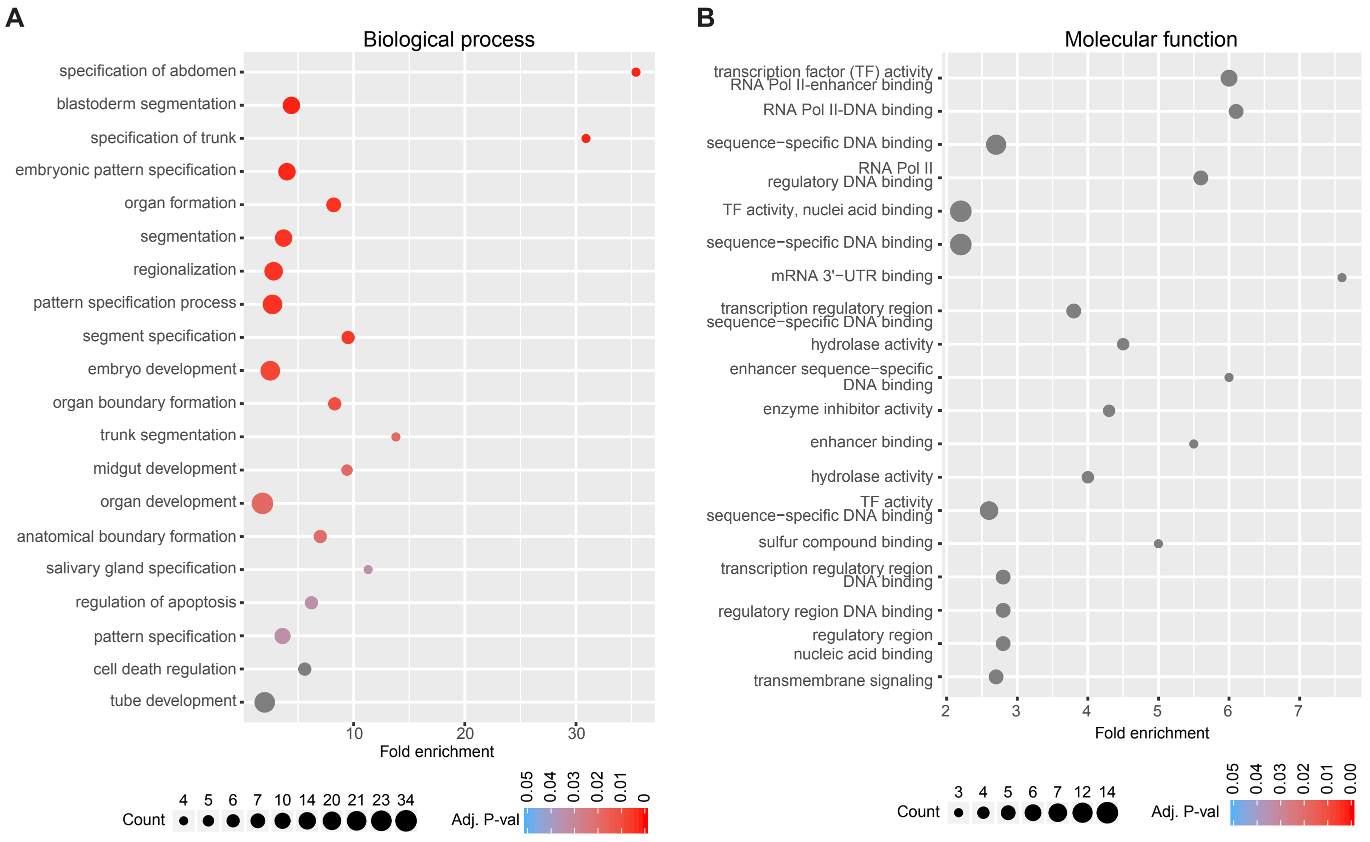

Figure S4. Functional enrichment of nuclear lncRNA-associated genes. Related to Figure 2
(A) GO term enrichment analysis of biological processes. (B) GO enrichment analysis of molecular functions. For each analysis, the first protein coding gene (PCG) neighbor at each side of the lncRNA was considered, while the entire high-quality set of PCGs expressed in our sample set was used as the reference. X-axis indicates fold enrichment between observed and expected GO terms, y-axis reports the significant biological process terms sorted by decreasing p-value. Dot size reflects the number of genes in that ontology, dot color indicates Benjamini-Hochberg adjusted p-values.

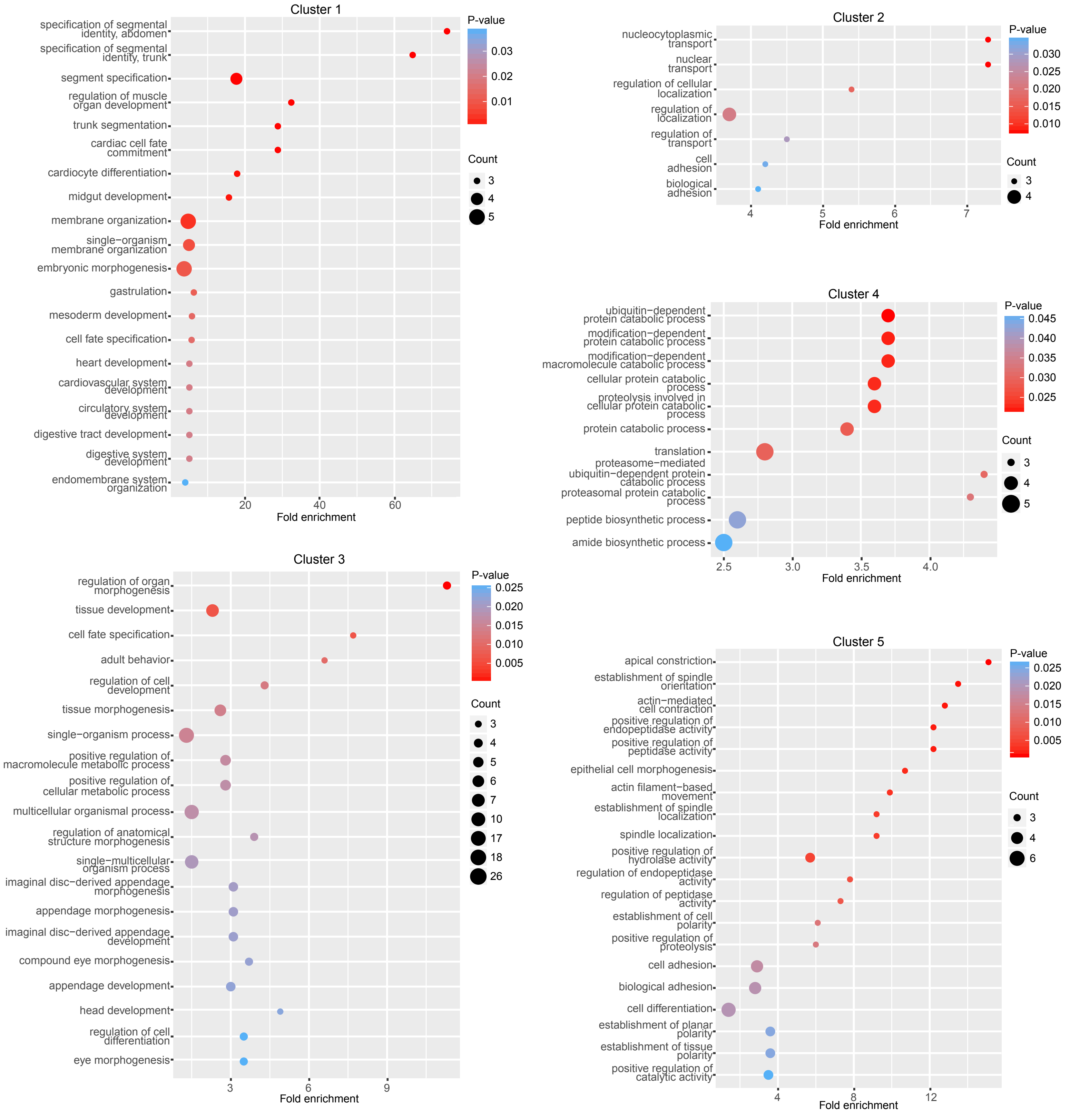

Figure S5. GO term enrichment of genes in the vicinity of lncRNA gene clusters. Related to Figure 2
Enrichment analysis of GO terms (biological processes) for the two closest protein coding gene (PCG) neighbors of lncRNA genes belonging to clusters 1 to 5. The high-quality set of PCGs expressed in our sample set was used as the reference. X-axis indicates fold enrichment between observed and expected GO terms, y-axis reports the significant biological process terms sorted by decreasing p-value. Dot size reflects the number of genes in that ontology, dot color indicates raw p-values.

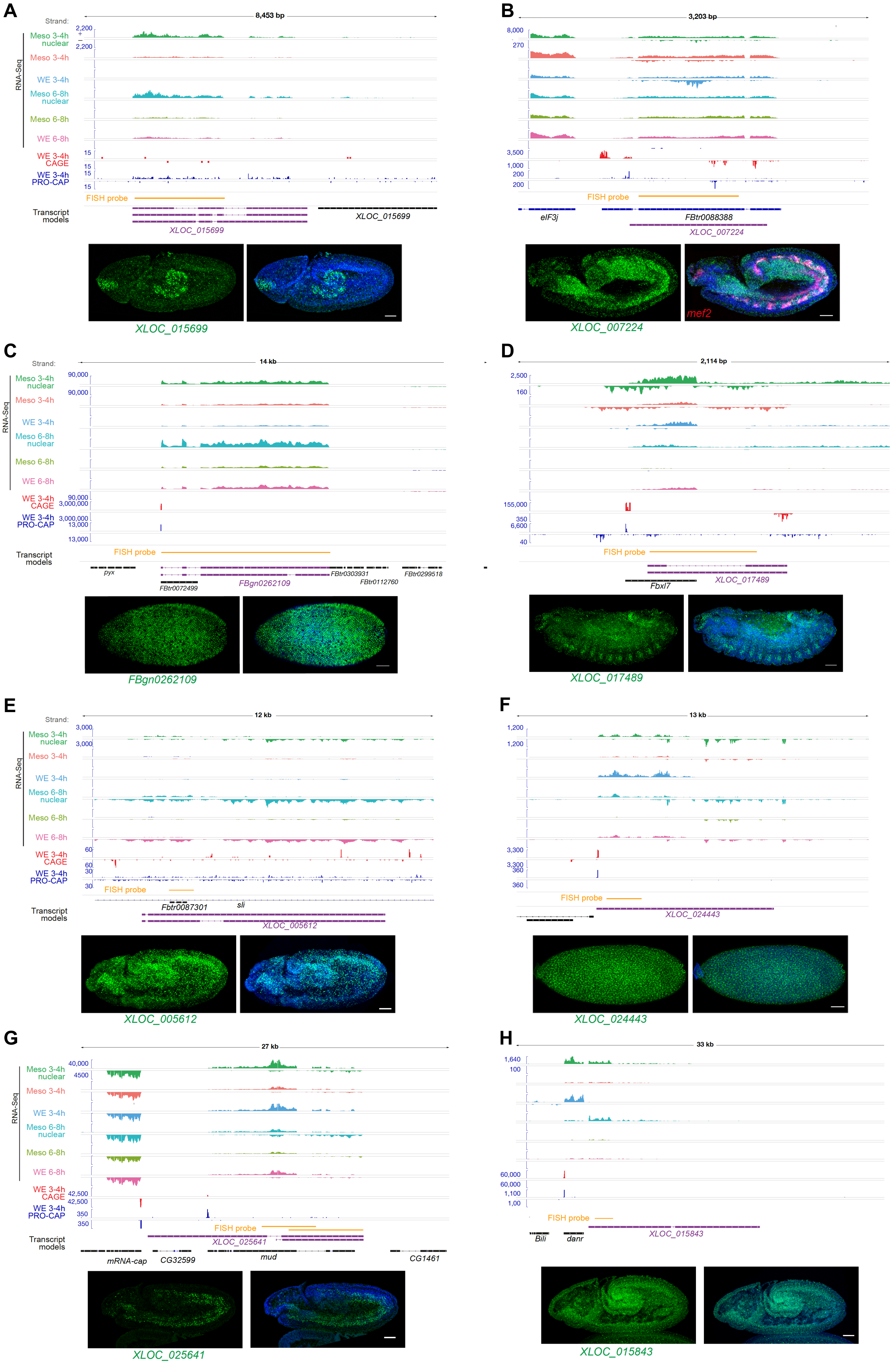

Figure S6. Temporal and Spatial expression of developmental lncRNAs. Related to Figure 2
(A-H) Above, genomic regions showing the expression of the indicated lncRNA gene (purple gene models) across samples. Meso = FACS purified mesodermal cells, WE = whole embryo, h = hours of embryogenesis. Below, fluorescent in situ hybridization (FISH) images of the lncRNA (green) with DAPI (blue) showing representative expression patterns. *XLOC_007224* is a double in-situ with a muscle marker (*Mef2*, red) (B). The coordinates and expression of all lncRNAs tested is provided in Table S5.

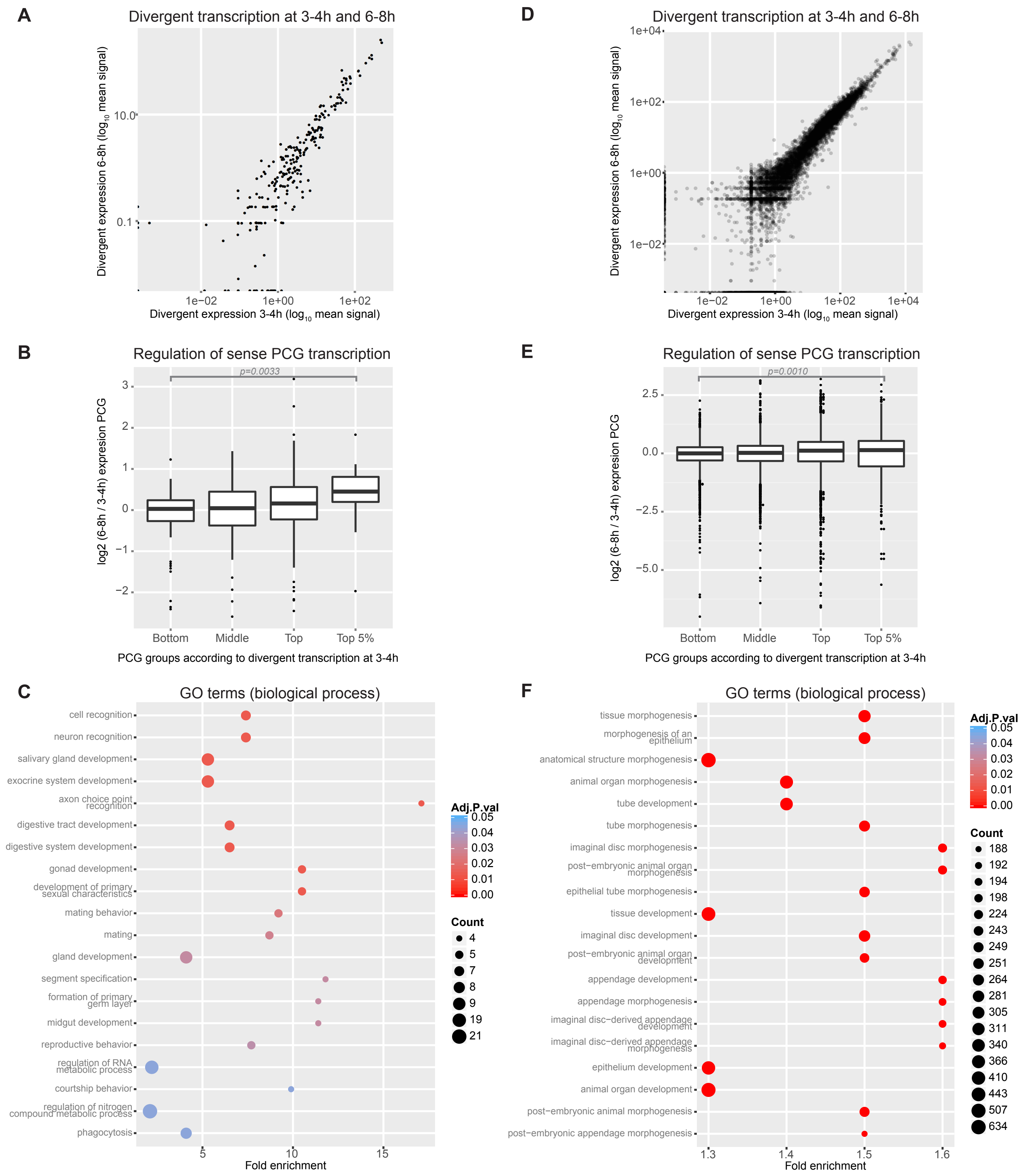

Figure S7. Analysis of divergent transcriptional units. Related to Figure 3

(A-C) lncRNA-associated PCG promoters (Figure 3C-D). (D-F) Promoters from all PCG active at mesoderm 3-4h (Figure 3E-F). (A, D) Divergent transcription is stable between 3-4h and 6-8h developmental times, as shown by the high correlation between expression values at both time-points (Pearson's r = 0.985 and 0.956 respectively). (B, E) Presence of divergent transcription predicts differential regulation of gene expression across developmental time for the PCG. Boxplots indicate change in expression from 3-4h to 6-8h of the PCGs correspondingto the different 3-4h divergent transcription groups. PCGs were divided in thirds and in addition the highest 5% is shown separately. P-value for Wilcoxon test is indicated. (C, F) Genes harboring divergent transcription (top third) are enriched in developmental functions. GO term enrichment analysis using the corresponding complete PCG set as universe (antisense lncRNA-associated PCG or all expressed in mesoderm at 3-4h.

Table S1. Overview of the transcriptome sequencing. Related to Figure 1

Summary of sequenced samples and mapped reads for the total RNA-seq and 5' CAGE analysis of gene expression on the twi::EGFP-CBP20 line.

Sample name	Origin	Library	Time point	Mapped reads
34_Sa	Mesoderm	total RNA-seq	3-4h	20121011
34_Sb	Mesoderm	total RNA-seq	3-4h	17504271
34_Sna	Mesoderm nuclei	total RNA-seq	3-4h	14580946
34_Snb	Mesoderm nuclei	total RNA-seq	3-4h	14883067
34_Ua	Whole embryo	total RNA-seq	3-4h	5055384
34_Ub	Whole embryo	total RNA-seq	3-4h	9050576
46_Sb	Mesoderm	total RNA-seq	4-6h	13402704
46_Sc	Mesoderm	total RNA-seq	4-6h	18569755
46_Ua	Whole embryo	total RNA-seq	4-6h	8769415
46_Ub	Whole embryo	total RNA-seq	4-6h	4181181
68_Sc	Mesoderm	total RNA-seq	6-8h	15781612
68_Sd	Mesoderm	total RNA-seq	6-8h	8877609
68_Snc	Mesoderm nuclei	total RNA-seq	6-8h	19991188
68_Snd	Mesoderm nuclei	total RNA-seq	6-8h	12754290
68_Ua	Whole embryo	total RNA-seq	6-8h	7058205
68_Uc	Whole embryo	total RNA-seq	6-8h	12176734
68_Ud	Whole embryo	total RNA-seq	6-8h	14744629
CAGE_U60_34h	Whole embryo	5' CAGE	3-4h	23241554
CAGE_S60_34h	Mesoderm	5' CAGE	3-4h	15876118
CAGE_U46_46h	Whole embryo	5' CAGE	4-6h	17738836
CAGE_S46_46h	Mesoderm	5' CAGE	4-6h	13564260
CAGE_U56_68h	Whole embryo	5' CAGE	6-8h	12576260
CAGE_S56_68h	Mesoderm	5' CAGE	6-8h	26555562
CAGE_meso_S13_r1	Mesoderm	5' CAGE	6-8h	24953370
CAGE_meso_S13_r2	Mesoderm	5' CAGE	6-8h	17308274
CAGE_meso_S3_r1	Mesoderm	5' CAGE	6-8h	38534497
CAGE_meso_S3_r2	Mesoderm	5' CAGE	6-8h	21603091

Table S6. Oligonucleotides used for FISH probe amplification and qPCR. Related to STAR Methods and Key Resources Table

Gene	Application	Fw Sequence (5' -> 3')	Rv Sequence (5' -> 3')
XLOC_010934	qPCR	GCCTGCAATCGTAAAGGATGG	TTTCGCACGGCTCTTGTTTC
XLOC_011009		AGCAAAAATCGCAGGCACAG	GCTGCAGCATGGAATTTTCC
XLOC_013478		TGGCAGACAACACACTTTCG	TTATTTCCCAACGGCCCTTG
FBgn0263019	cDNA amplification for FISH probe	CAAAAACGAGTCAGCGGCAA	ATGTGACTCCCGCTTTCGTT
FBgn0263595		GAAACCGAATGCGAATCCCG	ACTGGGCCATAAAGCAACCA
FBgn0266236		AGTGTCTGAATCACTGGGCG	TGGCTTTGACATTTCGTTCA
FBgn0266631		GGAAAAAGGATGCGAATCCGA	TCCTTGTTCAATCTAAGAGGCA
XLOC_004366		GGAAGGTATGGGATGGCCTG	GACGGATTTCGGAGTCGACA
XLOC_012225_1		GAATCCAAGGAGCGTGGTCA	TTGCCATTTCCATTGCAGCC
XLOC_012225_2		ATGCCCTGAAATCTTGCGGA	TAACGACGATCCAAGAGGGC
XLOC_012319		CCAGCCACGCATTTTGTCAA	TTGGCAGAGTGGGTGGTTTT
XLOC_018482		TAGAGCAGCGCGATAAAGCA	GAAGGACTTATCGGCCGTCG

Table S7. Comparison of our novel lncRNA genes set with the FlyBase r6.21 annotation. Related to STAR Methods

	Novel gene model	Transcripts in overlap	Annotated gene	Transcripts in overlap	Comments
Matching previously annotated transcripts	XLOC_004536	TCONS_00012691	CR46064	FBtr0347293	
	XLOC_005255	TCONS_00014942	CR45276	FBtr0345530	Novel model is longer
	XLOC_007166	TCONS_00020918	CR45321	FBtr0345669	
	XLOC_007956	TCONS_00023809	CR45270	FBtr0345480	Novel model is longer
		TCONS_00023810		FBtr0345792	
				FBtr0345793	
	XLOC_009721	TCONS_00028436	CR46005	FBtr0347134	Novel model is longer
		TCONS_00028437		FBtr0347135	
		TCONS_00028438			
		TCONS_00028439			
	XLOC_012319	TCONS_00036228	CR46003	FBtr0347130	
		TCONS_00036229			
		TCONS_00036230			
		TCONS_00036231			
		TCONS_00036232			
	XLOC_015145	TCONS_00043859	CR45631	FBtr0346325	
Previously annotated genes but new models may represent different transcript isoforms	XLOC_013181	TCONS_00038525	CR45912	FBtr0346984	Novel model reflects more accurately the read coverage
		TCONS_00038526		FBtr0346985	
				FBtr0346986	
	XLOC_024457	TCONS_00066961	flam	FBtr0347221	Overlap at the start region, but novel models include novel exonic region
		TCONS_00066962		FBtr0347222	
		TCONS_00066963		FBtr0347223	
		TCONS_00066964		FBtr0347224	
		TCONS_00066965		FBtr0347225	
		TCONS_00066966		FBtr0347226	
				FBtr0347227	
				FBtr0347467	
				FBtr0347468	
				FBtr0347469	
Different genes than those annotated	XLOC_000697	TCONS_00002074	CR46196	FBtr0347474	
		TCONS_00002075			
	XLOC_004996	TCONS_00014060	CR45309	FBtr0345583	
	XLOC_011009	TCONS_00031884	CR46216	FBtr0347511	
	XLOC_013478	TCONS_00039592	CR45966	FBtr0347079	
	XLOC_015885	TCONS_00046343	CR45651	FBtr0346368	
	XLOC_017217	TCONS_00050718	CR46016	FBtr0347170	
	XLOC_018845	TCONS_00055658	CR45573	FBtr0346231	
	XLOC_023269	TCONS_00062804	CR45519	FBtr0346055	
