# Supplementary material for: Non-coding RNA Expression, Function, and Variation during Drosophila Embryogenesis
Source: Curr Biol. 2018 Nov 19;28(22):3547–3561.e9. doi: 10.1016/j.cub.2018.09.026 (PMC6264527; doi:10.1016/j.cub.2018.09.026)
Supplement: Document S2. Article plus Supplemental Information [file mmc6.pdf]

Article

Current Biology

Non-coding RNA Expression, Function, and Variation during *Drosophila* Embryogenesis

Highlights

- Divergent lncRNA expression is positively correlated with highly expressed genes
- Despite complex expression, deletion of 3 lncRNAs had no obvious defects
- Natural sequence variation leads to non-coding intergenic transcription, i.e., lncRNA
- This could be used to identify recently evolved, as yet non-functional, lncRNAs

Authors

Ignacio E. Schor, Giovanni Bussotti, Matilda Maleš, Mattia Forneris, Rebecca R. Viales, Anton J. Enright, Eileen E.M. Furlong

Correspondence

furlong@embl.de

In Brief

Schor et al. identify hundreds of new lncRNAs expressed at specific embryonic stages and in mesoderm. Many have very specific spatiotemporal expression. Deletion of 3 lncRNAs are viable with no obvious phenotypes, even when stressed. Many lncRNAs have strain-specific expression, highlighting how sequence variation leads to intergenic transcription.

Check for updates

Schor et al., 2018, Current Biology *28*, 3547–3561
November 19, 2018 © 2018 The Author(s). Published by Elsevier Ltd.
https://doi.org/10.1016/j.cub.2018.09.026

CellPress

Current Biology
Article

Non-coding RNA Expression, Function, and Variation during *Drosophila* Embryogenesis

Ignacio E. Schor,[1,3,4] Giovanni Bussotti,[2,4,5] Matilda Maleš,[1,4,6] Mattia Forneris,[1] Rebecca R. Viales,[1] Anton J. Enright,[2,7] and Eileen E.M. Furlong[1,8,*]
[1]Genome Biology Unit, European Molecular Biology Laboratory (EMBL), 69117 Heidelberg, Germany
[2]European Molecular Biology Laboratory, European Bioinformatics Institute (EMBL-EBI), Hinxton CB10 1SD, UK
[3]Instituto de Fisiología, Biología Molecular y Neurociencias (IFIBYNE-CONICET), Departamento de Fisiología, Biología Molecular y Celular, FCEyN, Universidad de Buenos Aires, Buenos Aires, Argentina
[4]These authors contributed equally
[5]Present address: HUB Team, Institute Pasteur, Paris, France
[6]Present address: ZS, Frankfurt am Main, Frankfurt, Germany
[7]Present address: Department of Pathology, University of Cambridge, Cambridge, UK
[8]Lead Contact
*Correspondence: furlong@embl.de
https://doi.org/10.1016/j.cub.2018.09.026

SUMMARY

Long non-coding RNAs (lncRNAs) can often function in the regulation of gene expression during development; however, their generality as essential regulators in developmental processes and organismal phenotypes remains unclear. Here, we performed a tailored investigation of lncRNA expression and function during *Drosophila* embryogenesis, interrogating multiple stages, tissue specificity, nuclear localization, and genetic backgrounds. Our results almost double the number of annotated lncRNAs expressed at these embryonic stages. lncRNA levels are generally positively correlated with those of their neighboring genes, with little evidence of transcriptional interference. Using fluorescent *in situ* hybridization, we report the spatiotemporal expression of 15 new lncRNAs, revealing very dynamic tissue-specific patterns. Despite this, deletion of selected lncRNA genes had no obvious developmental defects or effects on viability under standard and stressed conditions. However, two lncRNA deletions resulted in modest expression changes of a small number of genes, suggesting that they fine-tune expression of non-essential genes. Several lncRNAs have strain-specific expression, indicating that they are not fixed within the population. This intra-species variation across genetic backgrounds may thereby be a useful tool to distinguish rapidly evolving lncRNAs with as yet non-essential roles.

INTRODUCTION

In addition to protein-coding genes, metazoan genomes contain many transcribed non-coding regions [1]. Among them, long non-coding RNAs (lncRNAs) represent a very heterogeneous group of non-coding transcripts, arbitrarily defined as having a transcript length of >200 bp with little or no evidence for coding potential [2]. Similar to mRNAs, lncRNAs are generally transcribed by RNA polymerase II (Pol II) and can therefore be spliced, capped, and polyadenylated [2, 3]. In comparison to protein-coding genes, genes encoding lncRNAs are more rapidly evolving [4–6] and tend to have more restricted expression in specific tissues [2, 7, 8] and developmental stages [9, 10].

Although extensive non-coding transcription of higher eukaryotes genomes is now widely recognized, whether and how most lncRNA molecules function is actively debated. The highly specific spatiotemporal expression patterns of many characterized lncRNAs are suggestive of function [11], although this could reflect bystander transcription during the regulation of tissue-specific protein-coding genes [12, 13]. Human genome-wide association studies (GWASs) suggest function for some lncRNAs by associating genetic variants disrupting lncRNA genes with specific traits [12, 14]. However, the relatively low stability of many lncRNAs, due to rapid exosome-mediated degradation, represents a strong argument against a possible function for the RNA molecule itself [15]. Although, even without accumulating to high levels, the transcription of some lncRNAs may affect expression of neighboring genes in *cis*, through mechanisms such as antisense-mediated repression [16, 17], RNA-mediated enhancement [18], activation of divergent genes in bidirectional promoters [19], and genomic imprinting [20].

Studies of individual lncRNAs identified functional roles in different biological processes, ranging from development and differentiation to cancer and metabolism [21–24]. Prominent examples of lncRNA involved in development include *Xist* essential for dosage compensation in mammals [25] and *rox1* and *rox2* essential for dosage compensation in *Drosophila* [26]. A large-scale effort to assess the function of lncRNA in mice revealed a lethal phenotype for three lncRNAs (out of eighteen deleted) and growth defects for another two [27], although possible effects of deleting regulatory elements contained within the deleted regions were not excluded [28]. The functional impact of other prominent lncRNAs during embryogenesis, such as the HOX-cluster-associated *Hotair* [29, 30], remains controversial [31] and involves considerations such as the absolute expression level of the lncRNA and affected genes in the

This is an open access article under the CC BY license (http://creativecommons.org/licenses/by/4.0/).

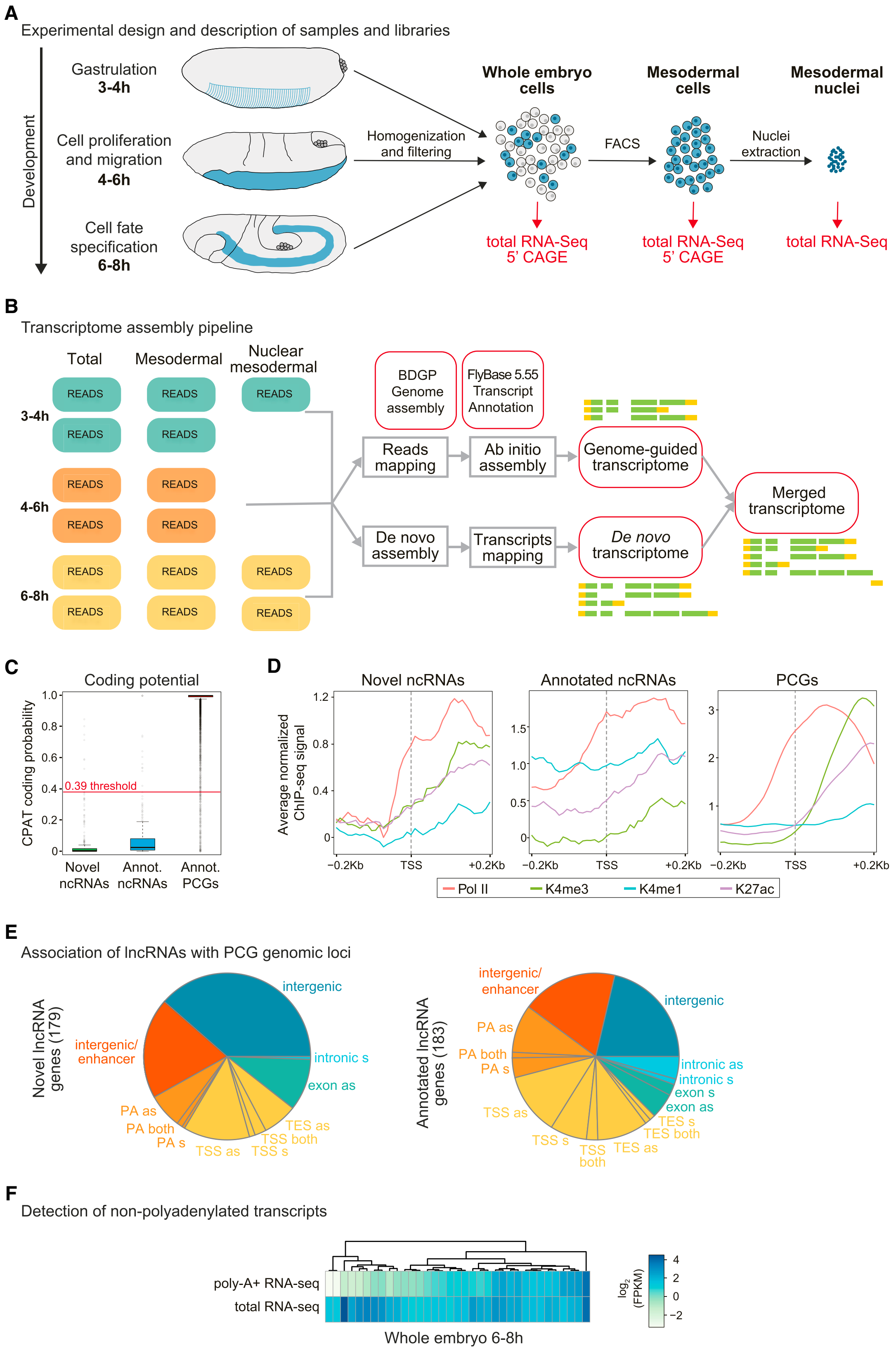

(legend on next page)

investigated tissues and the potential influence of genetic background [31, 32].

In *Drosophila*, although the function of some individual lncRNAs has been described [33–36], an integrative experimental approach that allows for the detection of most lncRNAs during specific stages of embryonic development is lacking. Previous genome-wide studies were primarily based on polyA+ RNA data [37–39]. However, as the efficiency of many RNA processing steps, including splicing and polyadenylation, is generally much lower for lncRNAs compared to mRNAs [15, 40], a non-polyA-based approach is needed to characterize the full repertoire of lncRNAs.

Here, by deeply sequencing rRNA-depleted total RNA at multiple stages of *Drosophila* embryogenesis, we roughly doubled the number of lncRNAs expressed at these specific embryonic stages. Our samples spanned stages from blastoderm to mid-embryogenesis, when major cell lineages are specified, and combined whole-embryo and tissue-specific analysis with cellular fractionation to enrich for nuclear transcripts. This complements previous lncRNA studies based on polyA+ RNA spanning stages throughout the entire life cycle [36, 39, 41, 42]. Half of our lncRNA set are differentially expressed across either developmental time or tissues, and 20% are enriched in nuclei. Using CRISPR/Cas9, we genetically deleted selected novel lncRNAs. In all cases, even though the lncRNAs had very specific spatio-temporal expression, they were not essential for embryonic development or viability under both standard and stressed conditions. The deletions had mild effects on gene expression, suggesting that, although not essential, these lncRNAs may play a role in fine-tuning gene expression. We also uncovered strain-specific differences in lncRNA expression, indicating that intra-species genetic variation can result in spurious non-coding transcription.

RESULTS

Identification of New Non-coding Transcripts during Embryonic Development

To obtain a comprehensive view of the transcriptional landscape during early and mid-stages of embryogenesis, we deeply sequenced rRNA-depleted total RNA samples from multiple developmental stages, cellular contexts (fluorescence-activated cell sorting [FACS]-sorted mesodermal cells [Meso] versus whole embryo [WE]), and subcellular compartments (nuclear RNA versus whole cell; Figure 1A; STAR Methods). The mesodermal samples were obtained by generating a transgenic *Drosophila* line with nuclear EGFP specifically expressed in mesoderm (under the control of a *twist* enhancer) [43]. Live embryos were dissociated into single cells and mesodermal cells isolated using FACS to greater than 95% purity (Meso; STAR Methods). These samples were complemented by stage-matched WE samples. To observe dynamic changes in lncRNA expression during embryogenesis, we sequenced paired Meso-WE samples from three different time intervals (Figure 1A): 3–4 hr (stages 6 and 7; spanning gastrulation and subsequent proliferation); 4–6 hr (stages 8 and 9; cell proliferation and migration); and 6–8 hr (stages 10 and 11; when there is substantial cell fate specification within the mesoderm and ectoderm). In addition, as we were particularly interested in RNA with a potential function in transcriptional regulation, we prepared RNA sequencing (RNA-seq) libraries from purified nuclear RNA from mesodermal cells at 3–4 hr and 6–8 hr. To more accurately determine the transcription start sites (TSSs) of the detected lncRNAs, we also prepared 5′ cap-analysis of gene expression (CAGE) libraries from Meso and WE samples at the three selected time points. Altogether, this resulted in 14 conditions (time, tissue, nuclear enrichment, and RNA-seq method), each with biological replicates (with the exception of the CAGE data; Figures 1A, S1A, and S1B; Table S1).

To obtain a comprehensive view of new transcripts, we applied a combination of *de novo* and reference-based transcript assembly (STAR Methods; Figure 1B), an approach previously shown to give more accurate and complete transcriptome assemblies [44]. Our assembled transcripts were subjected to differential expression analysis across stages and tissue (Figure S1C). In contrast to other lncRNA identification efforts [45–47], we did not require transcripts to be spliced but rather required a minimum length of 500 nt for monoexonic transcripts (in contrast to the standard 200 nt) and applied a series of strict filters to remove spurious, poorly supported, transcripts (Figure S2A; STAR Methods). This resulted in a high-confidence set of 179 novel genes, corresponding to 307 transcripts (Table S2). Applying the same filtering procedure to annotated *Drosophila* lncRNA genes (obtained from samples across many stages of embryogenesis, pupae, and adults) identified 183 genes (281 transcripts) that we consider actively transcribed at these stages of embryogenesis (Table S3). We also applied a similar approach to protein-coding genes (PCGs), resulting in a comparable set of transcripts: 8,227 PCGs and 16,658 transcripts that we consider robustly expressed during these stages (Table S4) and which

Figure 1. Identification of New lncRNAs during Embryonic Development

(A) Schematic overview of experimental design. Whole-embryo and mesodermal total RNA-seq stranded (100 bp paired-end) and 5′ CAGE (50 bp single-end) libraries were sequenced from 3–4 hr, 4–6 hr, and 6–8 hr embryos. Mesodermal nuclear RNA-seq libraries were prepared from 3–4 hr and 6–8 hr.

(B) Strategy overview of transcriptome assembly, combining *ab initio* and *de novo* assembly (STAR Methods).

(C) Novel lncRNAs lack coding potential. Boxplot showing CPAT coding potential predictions for our novel lncRNAs, previously annotated lncRNAs (FlyBase 5.55 annotation) and protein-coding genes (PCGs). Red line indicates threshold for coding potential (0.39).

(D) Histone modifications and RNA polymerase II (Pol II) presence at transcript start sites. Average chromatin immunoprecipitation sequencing (ChIP-seq) signal is shown for H3K27ac, H3K4me1, H3K4me3, and Pol II in mesoderm from 6–8 hr embryos [43], for promoter regions of novel and annotated lncRNAs and PCGs.

(E) Pie charts showing the genomic distribution of novel and annotated lncRNA genes with respect to PCGs. Genes are assigned to one class following the hierarchy: TSS > TES > exon > intron > promoter > enhancer > intergenic.

(F) Polyadenylation status of lncRNAs. Heatmaps show expression levels of novel lncRNAs in total RNA-seq (ribodepleted) and polyA-selected RNA-seq libraries from matched 6–8 hr whole-embryo samples.

See also Figures S2 and S3 and Tables S1, S2, S3, and S4.

were used for all subsequent analyses. Alignment of the novel transcripts to the latest genome build revealed that 9 of our 179 lncRNA genes (~5%) were included in the most recent annotation (currently containing 2,507 lncRNA genes; see STAR Methods). We have therefore compiled a comprehensive set of 362 lncRNA genes (192 annotated + 170 novel) that are confidently expressed during these stages of embryogenesis, which spans from blastoderm stages through to mid-embryogenesis. Almost half of these are described here for the first time, confirming the discovery value of our data and pipeline.

Characterization of Novel Developmental lncRNAs

We applied three complementary approaches to assess whether the novel transcripts are indeed likely to be non-coding (STAR Methods). First, we used coding potential assessment tool (CPAT) to score for coding potential [48]. Comparing the CPAT score of our novel transcripts to FlyBase annotated lncRNAs and PCGs expressed at the same stages (using the high-confident sets defined above) indicates that our novel transcripts have a very low coding potential, even lower than previously annotated lncRNAs (Figure 1C). Only 6 novel genes exceed the threshold of 0.39, calibrated for discriminating coding from non-coding transcripts in *D. melanogaster*. Second, BLAST we used to determine whether any predicted open reading frame (ORF) could correspond to known proteins or protein domains (Figure S2B). Third, we measured signatures of selection within the predicted ORFs across 12 *Drosophila* species using PhyloCSF [49] (Figure S2C). These analyses confirmed that the new transcripts have a coding capability comparable to that of annotated lncRNAs (Figures 1C, S2B, and S2C). To confidently predict individual instances of potentially coding genes, we required at least two of these methods to have values beyond threshold (see STAR Methods). Only 10 novel genes (out of 170) were positive for any two methods, and 3 were positive for all three methods. This is comparable to currently annotated *Drosophila* lncRNAs, where 8 high-quality (HQ) annotated genes (out of 183) were positive for any two methods and 2 were positive for all three methods. Therefore, the vast majority (~94%) of the newly identified genes likely correspond to novel lncRNAs, although we cannot exclude that some transcripts may encode micropeptides [50].

We also assessed whether novel lncRNAs might harbor primary microRNAs (miRNAs). Nucleotide BLAST was used to search for matches to known miRNAs, requiring both strands of the miRNA duplex to be on the same strand of the lncRNA gene, separated by a short region. Only two of our novel lncRNA genes show matches to known miRNA. Both occur on the opposite strand (Table S2), indicating that none of the lncRNA transcripts harbor primary miRNAs. In contrast, we identified nine lncRNAs within the currently annotated lncRNA set that harbor primary miRNAs (Table S3).

Using the RNA-seq signal and data from matching 5′ CAGE libraries (Figure S2C), we adjusted the start positions of 71 lncRNA transcripts to match a proximal 5′ CAGE peak (STAR Methods). These corrections improved the agreement of our annotated TSSs with independent indicators of gene start sites, such as mesoderm-specific accumulation of Pol II and chromatin modifications associated with active promoters [43], resulting in similar distributions to currently annotated *Drosophila* lncRNAs (Figure 1D). Although the average signal from CAGE and chromatin modifications gives the expected distribution, we observed extensive heterogeneity among individual lncRNA genes, with many, interestingly, lacking promoter-associated CAGE signal and/or histone modifications (Figure S3), a feature also observed in mammals [40].

Both novel and annotated lncRNAs are dispersed throughout the genome, including intergenic regions (>1 kb from a TSS), TSS or transcript end site (TES) overlapping, intron or exon overlapping, or promoter associated (PA), with respect to neighboring PCGs (Figure 1E). Interestingly, we detected a higher fraction of novel lncRNAs in intergenic regions compared to previously annotated lncRNAs (Figure 1E; p value = 0.000367; two-sided Fisher exact test), which may reflect a greater sequencing depth, the use of both *de novo* and reference-based assembly, and/or the ability to detect non-polyadenylated transcripts. To assess what fraction of the novel lncRNAs eluded previous detection due to a lack of polyadenylation, we compared the expression of our novel transcripts between total RNA-seq ribodepleted libraries from whole-embryo 6–8 hr samples to that of poly-A+ libraries generated from the same samples. 35 genes were detected as expressed (>2 reads per kilobase of transcript per million mapped reads [FPKM]) in the total RNA samples, almost half of which have lower expression levels in poly-A+ libraries, with a few being virtually undetectable (Figure 1F). This indicates that a large proportion of the novel intergenic lncRNAs is poorly poly-adenylated and is therefore generally not detected in standard polyA+ RNA-seq. This agrees with similar observation in human samples showing that lncRNA genes often show decreased poly-adenylation levels with respect to PCGs [2, 15].

Patterns of lncRNA Expression during Early Embryonic Development

To explore the general expression properties of embryonic lncRNAs, we combined our newly identified genes with the previously annotated lncRNAs that passed our expression filters, giving a comprehensive set of 362 non-coding genes expressed at these embryonic stages. Most lncRNAs have dynamic expression patterns (Figure 2A), with 52.2% being differentially expressed in at least one tested biological condition (excluding nuclear enrichment), in addition to 72 lncRNAs being significantly enriched in the nuclear fractions ($p < 0.01$; Benjamini-Hochberg adjusted p value). The closest PCGs in the vicinity of these nuclear-enriched transcripts are enriched for functions in basic developmental processes, such as segmentation, patterning, organ formation, and regionalization, including many genes involved in the regulation of transcription (Figure S4). Although this is consistent with a possible role of these nuclear transcripts in the *cis* regulation of early embryonic patterning genes, this may also reflect other phenomena, such as the sharing of regulatory elements acting on these processes.

To more formally assess the dynamic expression of the non-nuclear lncRNAs, we applied two rounds of *k*-means clustering (STAR Methods). This resulted in five robust clusters, containing 130 of the 362 lncRNA genes, with highly correlated expression during development (Figure 2B). Clusters 4 and 5 contain genes with expression enriched in mesoderm; cluster 4 transcripts are expressed at higher levels at the earlier two stages and then decrease, and cluster 5 transcripts increase as embryogenesis

CellPress

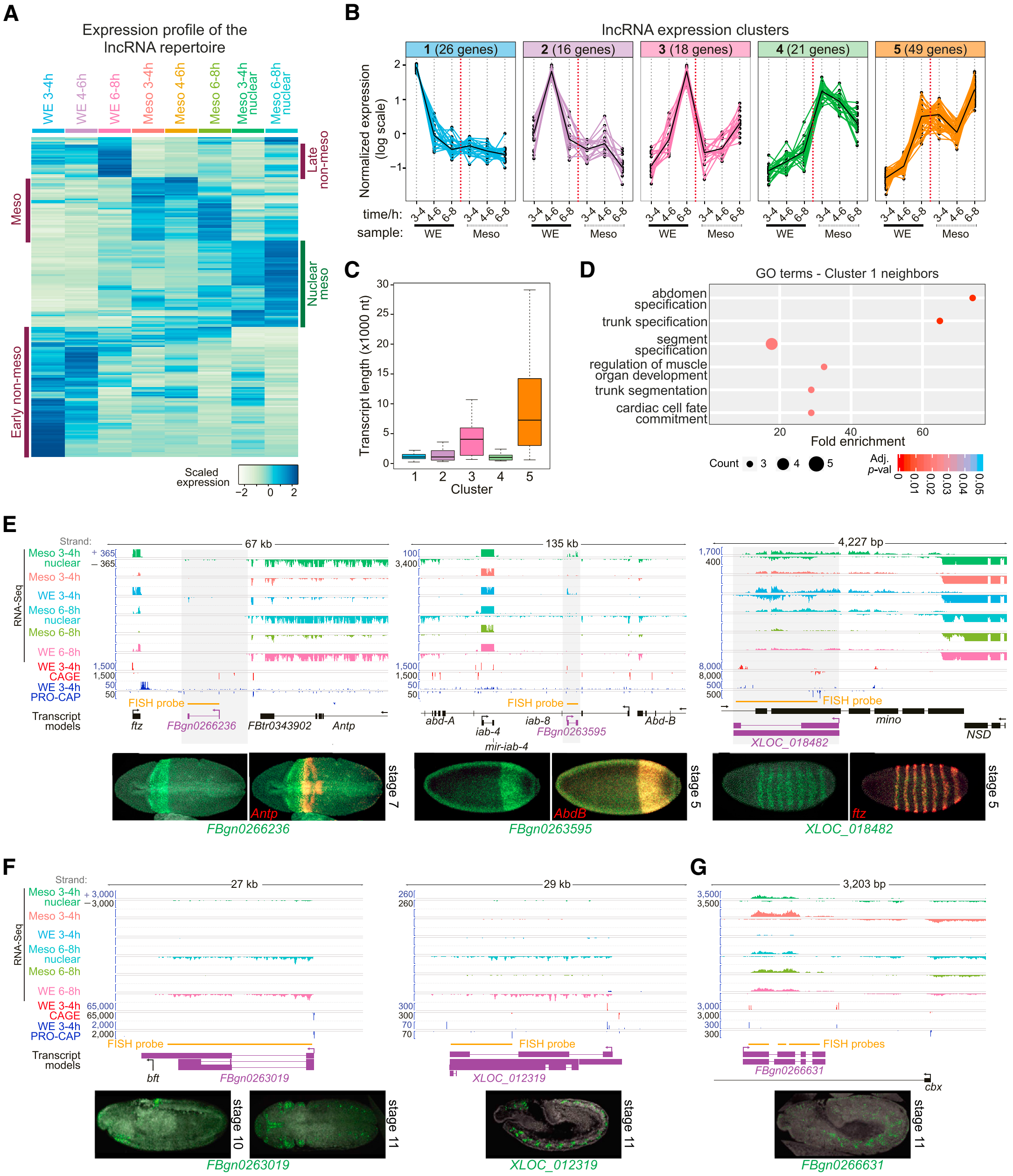

Figure 2. Expression Properties of Developmental lncRNAs

(A) Heatmap showing scaled expression levels of 362 lncRNA genes (both novel and annotated) across 8 conditions. Groups with mesodermal and nuclear over- or under-expression are indicated.

(B) Iterative *k*-means clustering (STAR Methods) identifies five robust groups of lncRNAs with highly correlated expression across conditions. x axis denotes the experimental conditions and y axis the normalized, scaled gene expression levels.

(C) Boxplot showing size distribution of transcripts in each expression cluster. Early clusters (1, 2, and 4) are significantly smaller than late clusters ($p = 1.294 \times 10^{-12}$; Wilcoxon test).

(D) Protein coding genes in the vicinity of early cluster 1 have functions in early embryo patterning. Dot plot shows GO biological process term enrichment for the two closest PCGs (one neighbor either side of each cluster 1 gene). x axis indicates fold enrichment between observed and expected and y axis the significant terms sorted by decreasing p value. Dot size reflects the number of genes in that ontology, and dot color indicates p value, corrected for multiple testing. Uncorrected p values for all significant terms in all clusters are shown in Figure S5.

(E–G) (Above) Genomic regions showing lncRNA expression (purple gene models) and their close neighbors (black gene models) across samples. The direction of transcription is indicated by reads above (sense) or below (antisense) the lines. Meso, mesoderm from FACS-purified cells; WE, whole embryo. (Below) Fluorescence *in situ* hybridization (FISH) images of lncRNA show early expression patterns (E), late expression patterns (F), or belonging to mesoderm-enriched set (G).

See also Figures S4, S5, and S6 and Table S5.

proceeds, being highest in the later time point (Figure 2B). Cluster 5 transcripts also increase expression in non-mesoderm tissues at 6–8 hr. Transcripts in these two clusters also differ in their median length, with cluster 5 transcripts being quite long (median of ~5.8 kb and some of ~30 kb; Figure 2C) and cluster 4 transcripts being among the shortest (median of ~1 kb; Figure 2C). Non-mesodermal transcripts, i.e., with higher expression in WE samples (clusters 1–3), have very dynamic stage-specific regulation (Figure 2B); expression of cluster 1 transcripts peak at 3–4 hr, cluster 2 transcripts peak at 4–6 hr, and cluster 3 transcripts have maximal expression (of the time points examined) at 6–8 hr. Similar to cluster 4, transcripts in clusters 1 and 2 are quite short (Figure 2C), mirroring the generally short transcripts observed for essential developmental genes at early stages of embryogenesis [51, 52]

We used the function of neighboring PCGs to assess the potential biological function of lncRNA genes within each cluster. After correction for multiple testing, only cluster 1 (very early genes) showed significant gene ontology (GO) term enrichments, which are mainly related to segment specification, suggesting a function in early embryonic patterning (Figure 2D). The remaining clusters likely contain genes with diverse function or not functionally associated with their neighboring PCGs (Figure S5).

We next assessed the spatiotemporal expression patterns of over 30 lncRNA genes (Table S5) by fluorescent *in situ* hybridization (FISH). FISH provides unique information about the lncRNAs' spatial and temporal pattern of expression and allows for direct comparison with the spatiotemporal pattern of the neighboring genes. Previous studies identified complex spatiotemporal expression, and heterogeneous sub-cellular distributions, for a number of lncRNA genes in *Drosophila* embryos [53]. Here, 16 out of the 30 lncRNAs tested gave a specific RNA FISH signal, 8 of which have very specific and restricted patterns of expression (shown in Figures 2, 4, and S6), although the expression of the remaining 8 was more diffuse or ubiquitous (summarized in Table S5). Expression of three lncRNAs (*FBgn0266236*, *FBgn0263595*, and *XLOC_018482*) was detected during early embryogenesis, two of which are within the early cluster 1 (*FBgn0263595* and *XLOC_018482*). These early transcripts show striking segmented patterns, which in two cases partially overlap that of the PCGs' expression in their vicinity (Figure 2E): *Antp* (lncRNA *FBgn0266236*) and *AbdB* (*FBgn0263595*). The expression pattern of *FBgn0266236* is similar to that previously described [53]. The third lncRNA (*XLOC_018482*) has an expression pattern resembling pair-rule genes, being detected in seven stripes at the blastoderm stage overlapping the expression of *ftz* (Figure 2E). As the *XLOC_018482* gene is located at a genomic position over 20 Mbp away from the *ftz* locus, its pair-rule expression is not readily explained by the regulation of *ftz* expression. Late non-mesodermal transcripts (*XLOC_012319* and *FBgn0263019*; the later included in cluster 3) were localized in ectodermal derivatives, such as the CNS, from stages 10 or 11 onward (Figure 2F). The mesodermally enriched transcript (*FBgn0266631*) has specific expression in the developing mesoderm at stages 10 or 11 (Figure 2G). Taken together, the highly specific spatiotemporal expression patterns of some lncRNA genes is suggestive of a function during embryogenesis, which we directly assess below.

The Relationship between Non-coding Transcription and Surrounding Gene Expression

We next investigated the relationship between lncRNA expression and the expression of their neighboring PCGs during embryogenesis and the plausibility of different mechanisms of *cis* regulation by sense and antisense lncRNA transcription. Each lncRNA was assigned to its closest neighbor (STAR Methods), forming lncRNA-PCG pairs that were used to evaluate the correlation in expression levels between (1) all lncRNAs and their closest neighbor (''closest neighbor,'' including overlapping genes; 395 pairs), (2) intergenic lncRNAs and their closest neighbor (''closest non-overlapping''; 234 pairs), (3) promoter proximal lncRNAs, not overlapping a PCG TSS (42 pairs), (4) antisense lncRNAs to a PCG exonic region, but not overlapping its TSS (29 pairs), and (5) antisense lncRNAs overlapping a PCG TSS (48 pairs), as depicted in Figure 3A. As a background control, we constructed a set of randomly assigned lncRNA-PCG pairs. We analyzed the distribution of correlation coefficients (considering expression across all samples, excluding the nuclear samples) for the different sets of pairs. Our results show a clear bias toward positive correlations between the expression of lncRNAs and their neighboring PCG when compared to random pairs, when all lncRNA or only intergenic lncRNA are taken into consideration (Figure 3B, left; Wilcoxon rank test: $p = 2.256 \times 10^{-7}$ and 6.987×10^{-7}, respectively). This suggests that, during embryonic development, the co-expression of lncRNA and neighboring PCG in the same tissues and/or stages are favored. Although this general positive association could suggest a role for lncRNAs in positively regulating transcription of their neighboring genes in *cis*, it is also in agreement with the proposal that close genes are co-regulated due to a shared *cis*-regulatory landscape [55]. In support of this, PCG-PCG gene pairs also show a positive correlation, both for the categories closest neighbor (Wilcoxon rank test: $p = 3.366 \times 10^{-34}$) and closest non-overlapping ($p = 1.778 \times 10^{-27}$). These results favor the general view of co-regulation of closest genes, similar to a bystander type of regulation.

lncRNA genes overlapping the promoter of their PCG neighbors, in either a sense or anti-sense orientation (Figure 3A), are not correlated with their expression (Figure 3B, right), but rather their distribution of correlation coefficients is close to random pairs (Wilcoxon rank test: $p = 0.944$). The same trend is observed when considering lncRNAs in close proximity to, but not overlapping, promoters of PCGs (Figures 3A and 3B; ''intergenic close to promoters''; $p = 0.729$). This lack of negative correlation argues against transcriptional silencing mediated by antisense transcription [16, 17] as a widespread mechanism in *Drosophila* embryonic development, although we note that such effects may be masked in measurements of steady-state RNA levels.

Divergent transcription of lncRNA-PCG pairs is frequent in mammals, and the expression levels of both genes co-vary during embryonic stem cell (ESC) differentiation [56]. In addition, the presence of a divergent lncRNA is often associated with the strong induction of transcription at the main sense promoter [19]. To specifically assess the impact of divergent transcription during *Drosophila* embryogenesis, we used our mesoderm-specific total RNA-seq datasets to limit potential heterogeneous signals coming from many cell types in the embryo. We first analyzed expression around the promoters of PCGs associated

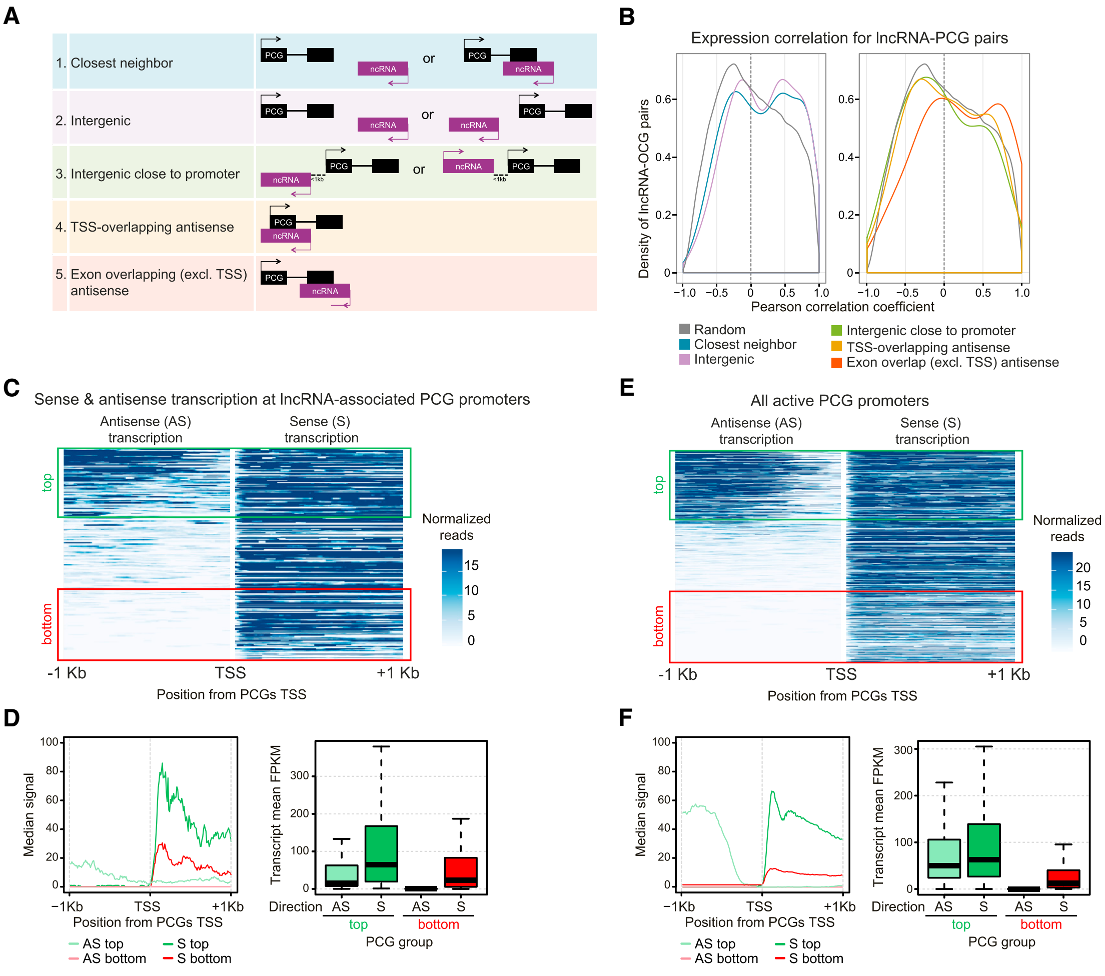

Figure 3. Expression of lncRNAs Is Correlated with Neighboring PCGs

(A) Schematic showing different lncRNA-PCG (protein coding gene) pair sets.

(B) Distribution of expression correlations between lncRNAs and their paired PCGs in each group (defined in A). Correlation coefficients calculated using expression levels across each individual sample are shown. Background set composed of 349,000 randomly associated lncRNA-PCG pairs (gray line) is shown.

(C) Sense and antisense (divergent) transcription around lncRNA-associated PCG promoters. PCGs involved in lncRNA-PCG antisense pairs considered from all groups (A) are shown: 501 lncRNA-PCG antisense pairs. Heatmap shows mesodermal RNA-seq (3–4 hr) counts of divergent transcripts (10-bp windows, across 2 kbp regions) centered on the TSS of annotated mesoderm-expressed PCG genes, in either antisense (left) or sense (right) direction. Transcripts ordered based on levels of antisense expression (divergent transcription) are shown. Top (green) and bottom (red) thirds of divergent expression are boxed.

(D) (Left) Median values of sense and antisense transcription for top and bottom groups. (Right) Genes with high (top third) divergent transcription have significantly higher expression levels than genes without (bottom third) divergent transcription ($p = 8.659e-09$; Wilcoxon test).

(E and F) Same as (C) and (D) but assessing promoters of all expressed PCGs (11,780 transcripts; STAR Methods). Again, genes with high (top third) divergent transcription have significantly higher expression levels than those with the low divergent transcription levels ($p < 2.2e-16$; Wilcoxon test; F).

See also Figure S7.

with an antisense lncRNA (gene pairs from groups 1–5; Figure 3A), centering on TSS of mesodermally expressed PCGs and ordering them according to their level of upstream antisense mesodermal total RNA-seq signal (Figure 3C). We performed the analysis at the 3–4 hr time point, but similar results are found at 6–8 hr as the levels of divergent transcription are highly correlated between both time points (Figure S7A). This analysis revealed an association between high levels of divergent lncRNA transcription with high levels of sense PCG expression (Figure 3C, compare top third in dark green versus bottom third in dark red; quantified in Figure 3D). In agreement with similar findings in mammals [19], the presence of divergent transcription at 3–4 hr predicted an increase in expression from the 3–4 hr to the 6–8 hr time interval (Figure S7B). The PCGs associated with high levels of divergent lncRNA transcription are significantly enriched in functions related to development (Figure S7C). Taken together, these results suggest that divergent lncRNA-PCG transcription could be a possible regulator of gene expression levels during embryonic development in *Drosophila*.

Although bidirectional promoters are generally not as prevalent in *Drosophila* as mammals [57], the findings above prompted us to analyze the full extent of divergent transcription during these embryonic stages. To assess this, we considered all PCGs active in a tissue (mesoderm) at a single time interval

(3–4 hr), irrespective of whether they have an annotated gene in a divergent configuration. Surprisingly, we detected high levels of antisense divergent transcription at about a third of genes, typically initiating within a 500-bp window upstream the PCG genes' TSS (Figure 3E) and expressed at both the 3–4 hr and 6–8 hr time points (Figure S7D). In agreement with lncRNA-associated PCGs, the expression level of genes with high levels of divergent transcription is significantly higher compared to genes without divergent transcription (Figures 3E and 3F; Wilcoxon test p value < 2.2e−16). Consistent with the previous results, the divergent architecture in *Drosophila* was associated with changes in the PCG's expression during these developmental stages and present at genes with a small but significant enrichment in processes related to development (Figures S7E and S7F). Therefore, the general properties of divergent transcriptional units are not limited to lncRNA-PCG pairs. This suggests either bystander gene expression for lncRNAs divergent to highly expressed PCGs and/or that the presence of divergent transcription enhances transcriptional output [19]. We confirmed the latter experimentally for one developmental lncRNA-PCG transcriptional unit at the *Doc1* locus (see below).

Assessing lncRNA Function through Genetic Deletion

To examine the function of lncRNAs in embryonic development, we selected three novel genes for targeted deletion using CRISPR/Cas9 and replaced the target region through homologous recombination with a DsRed selection marker [54] (Figure 4A; Table S5). The genes were selected after carefully screening genomic loci to ensure that a deletion could be made without disrupting characterized PCGs or developmental enhancers (see STAR Methods).

XLOC_012319, an ∼18-kb intergenic lncRNA gene (Figure 4B), is expressed at high levels in segmentally repeated neuronal precursors at the ventral midline at stages 9 or 10 and later in a subset of neurons overlapping *worniu* and *prospero* expression in the ventral nerve cord and brain (Figure 4C). The expression of this lncRNA partially overlaps the expression of *sim* and is detected in both the nucleus and cytoplasm (Figure 4C, lower panels). This very specific spatiotemporal expression and accumulation in the nucleus suggest a putative function in regulating the development of the embryonic nervous system. We deleted an ∼12.5-kb region, corresponding to more than half of the entire lncRNA gene, including its promoter and three DNaseI-hypersensitive sites (DHSs), to ensure that we would abolish its expression (Figure 4B). The knockout was confirmed by PCR using genomic DNA (not shown), RNA-seq (Figure 4B), and FISH (Figure 4D) from homozygous mutant embryos. Despite the gene's striking expression pattern, the lncRNA deletion had no effect on viability under normal laboratory conditions: the progeny of crosses between heterozygous parents followed expected Mendelian proportions, producing homozygous viable and fertile animals (Table 1). To assess a potential role of the lncRNA under stressful conditions, we challenged the knockout flies by placing heterozygous parents at 29°C but again failed to see any deviation from the expected proportions of mutant versus wild-type (WT) progeny (Table 1). Similarly, we placed both heterozygous and homozygous mutant flies separately under an extreme caloric restricted diet, housed in vials with only 1% agarose as their sole source of food, and observed a similar life expectancy of ∼4 days for both genotypes (not shown). The lncRNA *XLOC_012319* therefore appears to be a non-essential gene in terms of viability under laboratory conditions. We also did not observe differences in the development of neurons at these embryonic stages, as judged by immunofluorescence with a neuronal marker (Futsch; Figure 4D).

The second tested lncRNA gene, *XLOC_012225*, is located in a divergent orientation from *Doc1* (*Dorsocross1* [*FBgn0028789*]; Figure 4E), which codes for an essential transcription factor involved in amnioserosa differentiation, cardiogenesis, and the development of specific ectoderm derivatives [58–60]. *XLOC_012225* is an intron-containing lncRNA gene with a clear CAGE signal at its TSS (Figure 4E). Its divergent localization in proximity to an essential developmental regulator makes it an interesting candidate, given our observed positive correlation between divergent transcription and PCG expression (see Figures 3C–3F). Transcripts of *XLOC_012225* are detected in nuclei of dorsal ectoderm cells and amnioserosa (Figure 4F), in a pattern highly similar to *Doc1* [59, 60], indicating that the positive correlation between the two genes reflects their co-expression in the same cells as the same stages of embryogenesis. To obtain a knockout, we deleted a 1.25-kb region encompassing its TSS (Figure 4E) but did not remove known enhancers further downstream. As in the previous case, the homozygous deletion removing *XLOC_012225* transcription (Figure 4E) did not cause any observable effects on viability (Table 1). The spatial expression pattern of *Doc1* was also not obviously affected (Figure 4G), although we note there may be subtle quantitative changes in the levels of expression that are not detectable by *in situ* hybridization.

The third lncRNA selected, *XLOC_004366* (Figure 4H), is expressed in both Meso and WE samples, with high levels of expression at later stages. The gene is located in a heterochromatin-rich region, in a divergent orientation from a PCG (uncharacterized *CG12567* gene) about 2 kb away (Figure 4H). The lncRNA gene has genomic features of a typical mRNA gene, such as promoter-associated H3K4me3, Pol II, and CAGE peaks. *XLOC_004366* is detected at low levels by FISH, with an enrichment in the posterior endoderm primordium (Figure 4I). As in the two previous cases, the homozygous deletion, although it completely abolished the lncRNA's expression (Figures 4H and 4J) has no obvious effects on viability under normal and stress conditions (Table 1).

All three lncRNAs are therefore non-essential genes, at least regarding viability in laboratory conditions, although they may be required for additional functions that are essential for fitness in the wild. We note that non-essentiality does not necessarily mean non-functionality, because they may act redundantly or have more subtle roles under different conditions [61]. To assess whether these lncRNAs have a molecular phenotype that may not be apparent at the organismal level, we examined genome-wide expression using total RNA-seq from embryos homozygous for the deletion of each lncRNA at 6–8 hr after egg laying (AEL) (spanning stages 10 and 11), together with stage-matched embryos from the parental strain, as a control (Figure 5A). This time point was selected as the three candidate genes are expressed at these stages. Unfortunately, all independent collections sequenced for the *XLOC_004366* knockout (KO) line showed expression patterns with systematic biases that

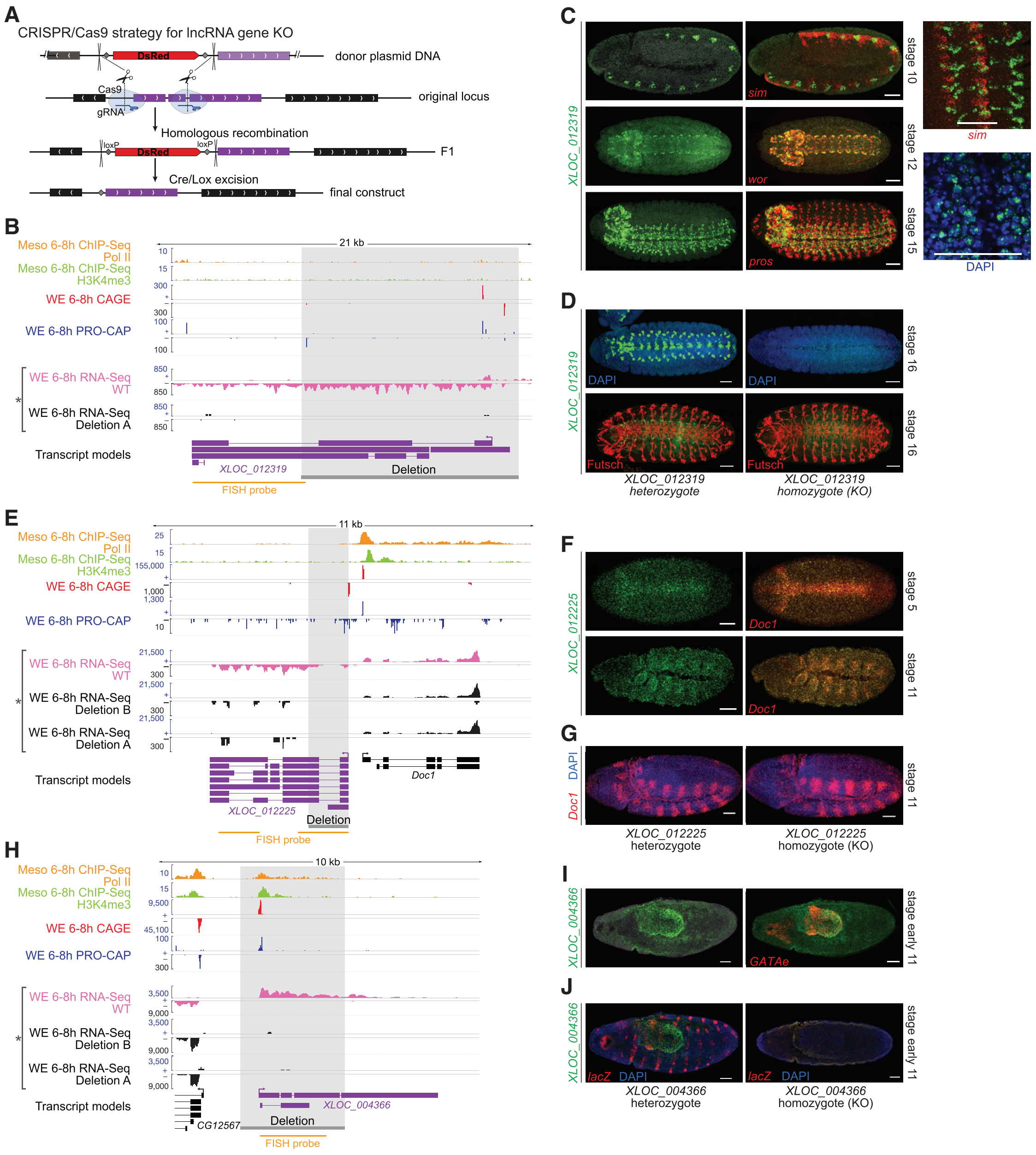

Figure 4. Assessing lncRNA Function by Gene Knockout

(A) Strategy for genetic deletions with CRISPR/Cas9 [54].

(B, E, and H) Genomic loci of *XLOC_012319* (B), *XLOC_012225* (E), and *XLOC_004366* (H) (purple; gene model) showing RNA-seq in WEs (pink track) and homozygous mutants from the deleted line (black track) at 6–8 hr, indicated by asterisk. Deleted region indicated by gray shading.

(C) Spatiotemporal expression of *XLOC_012319*. (Left panels) Double FISH of lncRNA (green; upper embryos) with different neuronal marker genes (red): ventral midline marker *single-minded* (*sim*); neuroblast marker *worniu* (*wor*); and ganglion mother cell (GMC) marker *prospero* (*pros*). (Right panels, upper) Zoomed image of *XLOC_012319* and *sim* expression shows co-expression in ventral midline. Signals do not overlap perfectly as *XLOC_012319* RNA is predominantly nuclear, shown by co-staining with DAPI.

(D) FISH of heterozygous and homozygous *XLOC_012319* embryos. *XLOC_012319* deletion completely abolishes lncRNA expression (left) but does not obviously affect neuronal development, as seen by immunofluorescence with an antibody against the neuronal marker Futsch (right).

(F) Double FISH of *XLOC_012225* lncRNA (green) and its divergent PCG, *Dorsocross-1* (*Doc1*, red). Left, stage 5 embryo, dorsal view, and right, stage 11 lateral view, show highly overlapping expression in dorsal ectoderm and amnioserosa.

(G) *XLOC_012225* KO (homozygous embryos) has normal *Doc1* expression.

(I) *XLOC_004366* is detected at low levels throughout the embryo and enriched in posterior endoderm primordium (green), marked by *GATAe* expression (red).

(J) lncRNA expression is undetectable in the homozygous mutant embryos (right). Heterozygous embryos were identified by *lacZ* expression from the balancer chromosome (red).

All scale bars represent 50 μm. See also Table 1.

Table 1. Viability Assessment of Knockout Lines

KO Line	Temperature (°C)	Homozygous Progeny	Heterozygous Progeny	Het/Hom Ratio
XLOC_004366 (a)	25	22	47	2.1
XLOC_004366 (a)	29	16	34	2.1
XLOC_004366 (b)	25	30	61	2.0
XLOC_004366 (b)	29	25	45	1.8
XLOC_012225 (a)	25	22	40	1.8
XLOC_012225 (a)	29	17	36	2.1
XLOC_012225 (b)	25	36	65	1.8
XLOC_012225 (b)	29	23	50	2.2
XLOC_012319	25	22	42	1.9
XLOC_012319	29	16	34	2.1

Siblings heterozygous KO stocks (deletion over balancer) were crossed at normal (25°C) or restrictive (29°C) temperatures, and the genotype of the progeny was evaluated using visible markers from the balancer chromosome. The expected proportions of adults if the KO is viable is 2/3 heterozygous (KO over balancer) and 1/3 homozygous KO, or a 2:1 Het/Hom ratio (homozygous balancer chromosomes are embryonic lethal).

prevented the analysis of this deletion. For the remaining two lncRNAs, each deletion was tested against the parental line and the other deletion.

Deletion of both *XLOC_012319* and *XLOC_012225* caused modest changes in expression of a relatively small number of genes (Figure 5B). *XLOC_012319* KO significantly affected the expression of 19 genes (adjusted p value < 0.05), which are distributed across the genome and have a median fold change of ~2.1. Six of these were overexpressed in the KO line, and the remaining 14 genes showed decreased expression levels. The expression of *XLOC_012319* is not restricted to one or two foci per nuclei (typical of nascent RNA at the transcribed locus) but rather accumulates throughout the nucleus (Figure 4C), consistent with a *trans*-acting role. We also noticed that *XLOC_012319* is located in a relatively gene-poor region, which is consistent with a lack of a *cis*-regulatory role. In contrast, *XLOC_012225* KO significantly affected 40 genes with a median fold change of ~2.2 (Figure 5B), with the majority having elevated expression, suggesting a repressive role. This deletion also showed a stronger local effect, including, for example, an ~30% reduction in the expression of the divergent *Doc1* gene (which was not detected by *in situ* hybridization) and an increase in expression of 10 other genes in a region of ±500 kb around the deletion by a median of ~1.5-fold (Figure 5C). This distinction suggests an involvement in *trans* and *cis* regulation for the two respective genes, although we cannot exclude mis-expression due to other, possibly secondary, effects.

Genotype-Dependent Changes in lncRNA Expression

As lncRNA expression is commonly tissue specific and rapidly evolving [4], we next examined lncRNA expression across two different genetic strains. To detect the lncRNAs described above, we used a *Drosophila* line (twi::EGFP) based on the Oregon R reference strain (Figure 1). To assess the impact of strain-specific differences, we compared RNA-seq data from this strain to a second strain (vas::Cas9), which was used for

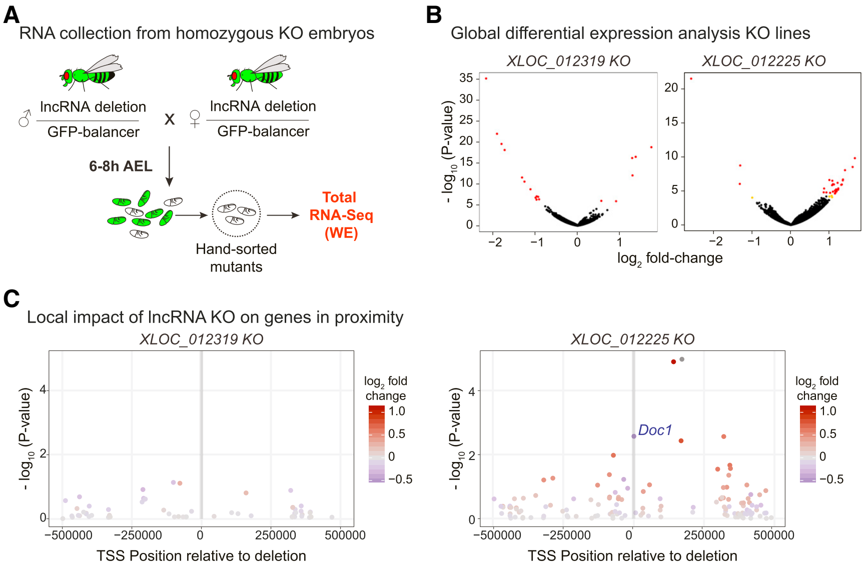

Figure 5. Differential Expression Analysis of KO Lines

(A) Scheme showing mutant embryo collection for RNA-seq at 6–8 hr after egg laying (AEL).

(B) Global differential expression changes: volcano plots show significantly affected genes for *XLOC_012319* and *XLOC_012225* knockout (KO) embryos, comparing two replicates from the KO against all other samples (including WT parental line and other mutants). Red dots depict genes with adjusted p value < 0.05, and orange dots indicate genes with sub-threshold significance but >2-fold change in mean expression.

(C) Local differential expression: raw p values (y axis) and log_2 fold change in expression (color scale) of genes located ±500,000 kbp from the center of the deletions (indicated by vertical gray boxes).

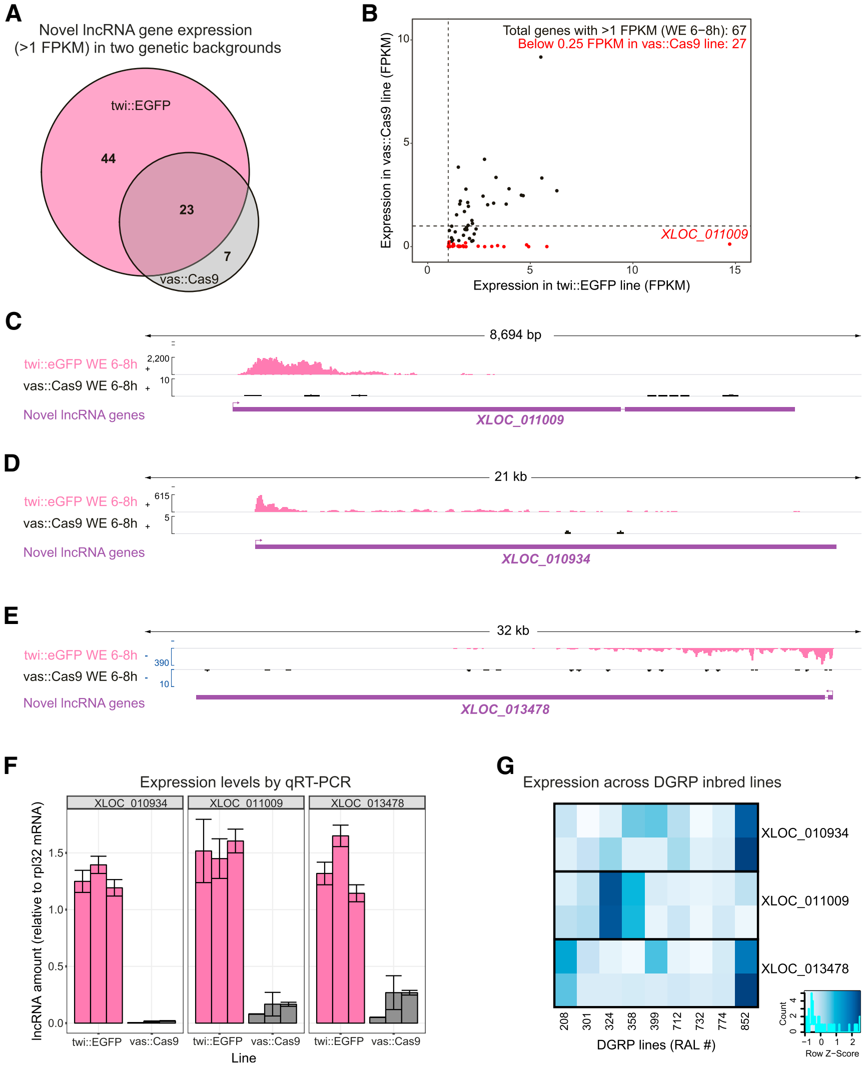

Figure 6. Detection of Strain-Specific Transcripts

(A) Venn diagram showing intersection between transcripts expressed (>1 FPKM) in two genetic backgrounds (twi::EGFP and vas::Cas9). Unique transcripts are biased toward the twi::EGFP line as the transcriptome assembly is based on this line.

(B) Expression values for the 67 lncRNA genes with expression >1 FPKM in the twi::EGFP background. Scatterplot indicates that 27 genes are virtually not expressed in vas::Cas9 line (red dots).

(C–E) *XLOC_011009* (C), *XLOC_010934* (D), and *XLOC_013478* (E) are examples of lncRNAs expressed in the twi::EGFP genetic background but completely absent at matching embryonic stages from the vas::Cas9 background.

(F) qRT-PCR confirming differences between twi::EGFP and vas::Cas9. Each bar represents an independent biological replicate; error bar is SE (from reaction duplicates).

(G) Assessment of strain-specific lncRNA expression across nine inbred *Drosophila* lines. Heatmap indicates qRT-PCR values. For each transcript line combination, the two tiles correspond to biological replicates.

the CRISPR-mediated deletions. We selected genes that are expressed in whole-embryo 6–8 hr Oregon R samples, using a relaxed threshold of 1 FPKM. Surprisingly, many lncRNA genes are expressed at levels below that threshold in the vas::Cas9 genetic background (Figure 6A), including 40% (27 out of 67) that are virtually undetectable (Figure 6B, red points).

Three examples of strain-specific lncRNAs that are expressed in Oregon R, but not in vas::Cas9, background are shown in Figures 6C–6E. All three lncRNAs have very prominent expression in Oregon R (twi::GFP) background but have little or no detectable expression in the vas::Cas9 background (Figures 6C–6E, compare pink to black track). We confirmed this strain specificity by qRT-PCR (Figure 6F), ruling out detection issues due to technical reasons, such as differences in sequencing depth. To extend this analysis to more genotypes, we analyzed the expression of these three lncRNA in nine inbred lines derived from wild isolates as part of the *Drosophila* genetic reference panel (DGRP) [62]. Expression of each of these strain-specific lncRNAs was detected in only 1 or 2 out of the 9 lines tested (Figure 6G).

These results indicate that a proportion of lncRNAs are very young and not fixed within the population, which suggests that they are non-functional despite their impressive expression. This scenario might be more common when looking at early developmental stages, particularly considering the proposed role of male reproductive organs as a source of novel genes during metazoan evolution [63]. In keeping with this, our lncRNA genes expressed at early embryonic stages have significantly lower conservation than those expressed constitutively or at later stages (median of phastCons scores 62, 169, and 195 for early, constitutive, and late, respectively; Mann-Whitney's p values of 0.0124 for early versus constitutive and 0.0179 for early versus late; STAR Methods). This trend is also observed if only intergenic transcripts are considered, eliminating possible confounding effects of lncRNA overlap to PCGs. In summary, these results highlight the importance of characterizing new transcripts in different genetic contexts (for example, in different strains or individuals within a population), which can help to identify newly evolving lncRNA genes.

DISCUSSION

Through deep sequencing of total RNA combined with extensive fluorescent *in situ* hybridization, we identified a comprehensive set of lncRNAs expressed at defined embryonic stages, many of which have specific spatiotemporal expression, in concordance with reports in other contexts [53]. lncRNA transcription is generally correlated with the expression of neighboring PCGs during development. This, in addition to the fact that many lncRNAs recapitulate part of their neighboring gene's expression, suggests that they share chromatin domains or

regulatory elements with developmental genes. In some cases, the lncRNA transcripts may even be generated from the regulatory elements, due to enhancer transcription [12, 13], for example. lncRNAs located in a divergent position from the promoter of expressed PCGs appear to be a more extreme case, where the levels of lncRNA expression scale with the levels of the divergent PCGs. At least for one case (*XLOC_012225*), we showed that deletion of the lncRNA caused a partial decrease (~30%) in the divergent PCG gene's expression, *Doc1* (Figure 4E). This may be an example of a lncRNA gene that is currently non-essential but might be in the process of being co-opted for a regulatory function and becoming a stabilized transcriptional unit.

Despite having very interesting and spatially restricted expression, genetic deletion of three lncRNAs showed no obvious developmental defects and no requirement for viability under normal and stressed conditions. This lack of strong phenotypes is consistent with recent findings for new genes in *Drosophila* [64], although novel genes and lncRNA genes in particular seem to frequently affect male fertility [36, 64]. It is interesting to note that these findings based on genetic knockout are in apparent contrast to previous reports of widespread effects on viability using RNAi to knock down lncRNA expression [65]. This suggests that the disruption approach may influence the observations, as observed comparing genetic deletions and morpholinos in zebrafish [66]. The lack of phenotypes in our case may also reflect the stages of embryogenesis that we focused on. lncRNA expression appears more pervasive in certain differentiated tissues, such as the male reproductive system [2] and nervous system [67], and may therefore play a more prominent role during these later stages. This is in keeping with the lower conservation we observed for lncRNAs specifically expressed at early embryonic stages.

Interestingly, our results revealed that a significant number of lncRNAs, often with very robust and complex expression, are only expressed in a strain-specific manner. This implies that these transcriptional units are not stabilized within the population, arguing against an essential function during embryogenesis. Although they represent an interesting class of genes with very recent evolution, they also highlight the need for caution in the interpretation of lncRNA function from expression studies performed in just one genetic background.

STAR★METHODS

Detailed methods are provided in the online version of this paper and include the following:

- KEY RESOURCES TABLE
- CONTACT FOR REAGENT AND RESOURCE SHARING
- EXPERIMENTAL MODEL AND SUBJECT DETAILS
  - ○ Fly line for isolation of mesodermal cells via FACS
  - ○ Generation of lncRNA deletion using CRISPR/Cas9
  - ○ Strategy for collection of knockout embryos for RNA-Seq
- METHOD DETAILS
  - ○ Sample collection, fluorescence activated cell sorting and RNA isolation
  - ○ Assessment of mesodermal enrichment after FACS
  - ○ Preparation of nuclear extracts from sorted live cells
  - ○ Depletion of rRNA for total RNA-seq
  - ○ RNA-seq library preparation and sequencing
  - ○ 5′ CAGE library preparation
  - ○ Double fluorescent *in situ* hybridization
  - ○ Viability assays for transgenic flies
  - ○ Quantification of lncRNA expression levels by qRT-PCR
- QUANTIFICATION AND STATISTICAL ANALYSIS
  - ○ Mapping and assembling pipeline
  - ○ Filtering pipeline and differential expression analysis
  - ○ Comparison with the latest genome annotation
  - ○ Coding potential analysis
  - ○ Improving the annotation of the 5′ end of the lncRNA by TSS clipping and extension
  - ○ Histone mark and pol II support of mesodermal promoters
  - ○ 5′ CAGE mapping and support of transcription start sites
  - ○ Ribodepleted versus Poly-A+ selected libraries:
  - ○ lncRNA classification
  - ○ lncRNA-PCG expression correlation
  - ○ Expression clusters
  - ○ GO analysis
  - ○ Bidirectional transcription from PCG promoters
  - ○ Differential expression analysis of the KO lines
  - ○ Genotype specific variation in lncRNA expression
  - ○ Conservation of lncRNA genes expressed at different stages
- DATA AND SOFTWARE AVAILABILITY
- ADDITIONAL RESOURCES

SUPPLEMENTAL INFORMATION

Supplemental Information includes seven figures and seven tables and can be found with this article online at https://doi.org/10.1016/j.cub.2018.09.026.

ACKNOWLEDGMENTS

We are very grateful to all members of the Furlong lab for discussions and comments, especially James Reddington, Tim Pollex, Olga Mikhaylichenko, and Nezha Benabdallah. We thank Guillaume Junion and Hilary Gustafson for generating the twist::EGFP-CBP20 line. This work was technically supported by the EMBL Genomics, Flow Cytometry, and Advanced Light Microscopy Core Facilities and the public resources of FlyBase, BDGP, and RedFly. The work was financially supported by the FP7 European Research Council (ERC) grant agreement 322851 (ERC advanced grant CisRegVar) to E.E.M.F., EU FP7 Marie Curie actions (299504, ncRNAsDevNet) to I.E.S., EMBL-cofund EIPOD programme to G.B., and the BBSRC (BB/J01589X/1) to A.J.E.

AUTHOR CONTRIBUTIONS

Conceptualization, I.E.S., G.B., M.M., A.J.E., and E.E.M.F.; Methodology, I.E.S., M.M., and R.R.V.; Software, G.B.; Formal Analysis, G.B., M.F., and I.E.S.; Investigation, M.M., I.E.S., G.B., R.R.V., M.F., and E.E.M.F.; Writing – Original Draft, I.E.S., G.B., M.M., and E.E.M.F.; Writing – Review & Editing, I.E.S., G.B., M.M., A.J.E., and E.E.M.F.; Supervision, A.J.E. and E.E.M.F.; Funding Acquisition, I.E.S., A.J.E., and E.E.M.F.

DECLARATION OF INTERESTS

The authors declare no competing interests.

Received: March 14, 2018
Revised: July 10, 2018
Accepted: September 12, 2018
Published: November 1, 2018

REFERENCES

1. Dunham, I., Kundaje, A., Aldred, S.F., Collins, P.J., Davis, C.A., Doyle, F., Epstein, C.B., Frietze, S., Harrow, J., Kaul, R., et al.; ENCODE Project Consortium (2012). An integrated encyclopedia of DNA elements in the human genome. Nature *489*, 57–74.
2. Derrien, T., Johnson, R., Bussotti, G., Tanzer, A., Djebali, S., Tilgner, H., Guernec, G., Martin, D., Merkel, A., Knowles, D.G., et al. (2012). The GENCODE v7 catalog of human long noncoding RNAs: analysis of their gene structure, evolution, and expression. Genome Res. *22*, 1775–1789.
3. Quinn, J.J., and Chang, H.Y. (2016). Unique features of long non-coding RNA biogenesis and function. Nat. Rev. Genet. *17*, 47–62.
4. Kutter, C., Watt, S., Stefflova, K., Wilson, M.D., Goncalves, A., Ponting, C.P., Odom, D.T., and Marques, A.C. (2012). Rapid turnover of long noncoding RNAs and the evolution of gene expression. PLoS Genet. *8*, e1002841.
5. Quinn, J.J., Zhang, Q.C., Georgiev, P., Ilik, I.A., Akhtar, A., and Chang, H.Y. (2016). Rapid evolutionary turnover underlies conserved lncRNA-genome interactions. Genes Dev. *30*, 191–207.
6. Ulitsky, I., and Bartel, D.P. (2013). lincRNAs: genomics, evolution, and mechanisms. Cell *154*, 26–46.
7. Cabili, M.N., Trapnell, C., Goff, L., Koziol, M., Tazon-Vega, B., Regev, A., and Rinn, J.L. (2011). Integrative annotation of human large intergenic noncoding RNAs reveals global properties and specific subclasses. Genes Dev. *25*, 1915–1927.
8. Washietl, S., Kellis, M., and Garber, M. (2014). Evolutionary dynamics and tissue specificity of human long noncoding RNAs in six mammals. Genome Res. *24*, 616–628.
9. Batista, P.J., and Chang, H.Y. (2013). Long noncoding RNAs: cellular address codes in development and disease. Cell *152*, 1298–1307.
10. Flynn, R.A., and Chang, H.Y. (2014). Long noncoding RNAs in cell-fate programming and reprogramming. Cell Stem Cell *14*, 752–761.
11. Ulitsky, I., Shkumatava, A., Jan, C.H., Sive, H., and Bartel, D.P. (2011). Conserved function of lincRNAs in vertebrate embryonic development despite rapid sequence evolution. Cell *147*, 1537–1550.
12. Hon, C.C., Ramilowski, J.A., Harshbarger, J., Bertin, N., Rackham, O.J., Gough, J., Denisenko, E., Schmeier, S., Poulsen, T.M., Severin, J., et al. (2017). An atlas of human long non-coding RNAs with accurate 5′ ends. Nature *543*, 199–204.
13. Mikhaylichenko, O., Bondarenko, V., Harnett, D., Schor, I.E., Males, M., Viales, R.R., and Furlong, E.E.M. (2018). The degree of enhancer or promoter activity is reflected by the levels and directionality of eRNA transcription. Genes Dev. *32*, 42–57.
14. Guo, H., Ahmed, M., Zhang, F., Yao, C.Q., Li, S., Liang, Y., Hua, J., Soares, F., Sun, Y., Langstein, J., et al. (2016). Modulation of long noncoding RNAs by risk SNPs underlying genetic predispositions to prostate cancer. Nat. Genet. *48*, 1142–1150.
15. Schlackow, M., Nojima, T., Gomes, T., Dhir, A., Carmo-Fonseca, M., and Proudfoot, N.J. (2017). Distinctive patterns of transcription and RNA processing for human lincRNAs. Mol. Cell *65*, 25–38.
16. Camblong, J., Iglesias, N., Fickentscher, C., Dieppois, G., and Stutz, F. (2007). Antisense RNA stabilization induces transcriptional gene silencing via histone deacetylation in S. cerevisiae. Cell *131*, 706–717.
17. Hongay, C.F., Grisafi, P.L., Galitski, T., and Fink, G.R. (2006). Antisense transcription controls cell fate in Saccharomyces cerevisiae. Cell *127*, 735–745.
18. Ørom, U.A., Derrien, T., Beringer, M., Gumireddy, K., Gardini, A., Bussotti, G., Lai, F., Zytnicki, M., Notredame, C., Huang, Q., et al. (2010). Long noncoding RNAs with enhancer-like function in human cells. Cell *143*, 46–58.
19. Scruggs, B.S., Gilchrist, D.A., Nechaev, S., Muse, G.W., Burkholder, A., Fargo, D.C., and Adelman, K. (2015). Bidirectional transcription arises from two distinct hubs of transcription factor binding and active chromatin. Mol. Cell *58*, 1101–1112.
20. Latos, P.A., Pauler, F.M., Koerner, M.V., Şenergin, H.B., Hudson, Q.J., Stocsits, R.R., Allhoff, W., Stricker, S.H., Klement, R.M., Warczok, K.E., et al. (2012). Airn transcriptional overlap, but not its lncRNA products, induces imprinted Igf2r silencing. Science *338*, 1469–1472.
21. Fatica, A., and Bozzoni, I. (2014). Long non-coding RNAs: new players in cell differentiation and development. Nat. Rev. Genet. *15*, 7–21.
22. Bergmann, J.H., Li, J., Eckersley-Maslin, M.A., Rigo, F., Freier, S.M., and Spector, D.L. (2015). Regulation of the ESC transcriptome by nuclear long noncoding RNAs. Genome Res. *25*, 1336–1346.
23. Schmitt, A.M., and Chang, H.Y. (2016). Long noncoding RNAs in cancer pathways. Cancer Cell *29*, 452–463.
24. Schmitz, S.U., Grote, P., and Herrmann, B.G. (2016). Mechanisms of long noncoding RNA function in development and disease. Cell. Mol. Life Sci. *73*, 2491–2509.
25. Ballabio, A., and Willard, H.F. (1992). Mammalian X-chromosome inactivation and the XIST gene. Curr. Opin. Genet. Dev. *2*, 439–447.
26. Ilik, I., and Akhtar, A. (2009). roX RNAs: non-coding regulators of the male X chromosome in flies. RNA Biol. *6*, 113–121.
27. Sauvageau, M., Goff, L.A., Lodato, S., Bonev, B., Groff, A.F., Gerhardinger, C., Sanchez-Gomez, D.B., Hacisuleyman, E., Li, E., Spence, M., et al. (2013). Multiple knockout mouse models reveal lincRNAs are required for life and brain development. eLife *2*, e01749.
28. Bassett, A.R., Akhtar, A., Barlow, D.P., Bird, A.P., Brockdorff, N., Duboule, D., Ephrussi, A., Ferguson-Smith, A.C., Gingeras, T.R., Haerty, W., et al. (2014). Considerations when investigating lncRNA function in vivo. eLife *3*, e03058.
29. Li, L., Liu, B., Wapinski, O.L., Tsai, M.C., Qu, K., Zhang, J., Carlson, J.C., Lin, M., Fang, F., Gupta, R.A., et al. (2013). Targeted disruption of Hotair leads to homeotic transformation and gene derepression. Cell Rep. *5*, 3–12.
30. Rinn, J.L., Kertesz, M., Wang, J.K., Squazzo, S.L., Xu, X., Brugmann, S.A., Goodnough, L.H., Helms, J.A., Farnham, P.J., Segal, E., and Chang, H.Y. (2007). Functional demarcation of active and silent chromatin domains in human HOX loci by noncoding RNAs. Cell *129*, 1311–1323.
31. Amândio, A.R., Necsulea, A., Joye, E., Mascrez, B., and Duboule, D. (2016). Hotair is dispensible for mouse development. PLoS Genet. *12*, e1006232.
32. Selleri, L., Bartolomei, M.S., Bickmore, W.A., He, L., Stubbs, L., Reik, W., and Barsh, G.S. (2016). A Hox-embedded long noncoding RNA: is it all hot air? PLoS Genet. *12*, e1006485.
33. Mulvey, B.B., Olcese, U., Cabrera, J.R., and Horabin, J.I. (2014). An interactive network of long non-coding RNAs facilitates the Drosophila sex determination decision. Biochim. Biophys. Acta *1839*, 773–784.
34. Petruk, S., Sedkov, Y., Riley, K.M., Hodgson, J., Schweisguth, F., Hirose, S., Jaynes, J.B., Brock, H.W., and Mazo, A. (2006). Transcription of bxd noncoding RNAs promoted by trithorax represses Ubx in cis by transcriptional interference. Cell *127*, 1209–1221.
35. MNutt, M. (2014). Retraction. Science *344*, 981.
36. Wen, K., Yang, L., Xiong, T., Di, C., Ma, D., Wu, M., Xue, Z., Zhang, X., Long, L., Zhang, W., et al. (2016). Critical roles of long noncoding RNAs in Drosophila spermatogenesis. Genome Res. *26*, 1233–1244.
37. Graveley, B.R., Brooks, A.N., Carlson, J.W., Duff, M.O., Landolin, J.M., Yang, L., Artieri, C.G., van Baren, M.J., Boley, N., Booth, B.W., et al. (2011). The developmental transcriptome of Drosophila melanogaster. Nature *471*, 473–479.
38. Young, R.S., Marques, A.C., Tibbit, C., Haerty, W., Bassett, A.R., Liu, J.-L., and Ponting, C.P. (2012). Identification and properties of 1,119 candidate lincRNA loci in the Drosophila melanogaster genome. Genome Biol. Evol. *4*, 427–442.

39. Brown, J.B., Boley, N., Eisman, R., May, G.E., Stoiber, M.H., Duff, M.O., Booth, B.W., Wen, J., Park, S., Suzuki, A.M., et al. (2014). Diversity and dynamics of the Drosophila transcriptome. Nature *512*, 393–399.
40. Melé, M., Mattioli, K., Mallard, W., Shechner, D.M., Gerhardinger, C., and Rinn, J.L. (2017). Chromatin environment, transcriptional regulation, and splicing distinguish lincRNAs and mRNAs. Genome Res. *27*, 27–37.
41. Chen, B., Zhang, Y., Zhang, X., Jia, S., Chen, S., and Kang, L. (2016). Genome-wide identification and developmental expression profiling of long noncoding RNAs during Drosophila metamorphosis. Sci. Rep. *6*, 23330.
42. Nyberg, K.G., and Machado, C.A. (2016). Comparative expression dynamics of intergenic long noncoding RNAs in the genus Drosophila. Genome Biol. Evol. *8*, 1839–1858.
43. Bonn, S., Zinzen, R.P., Girardot, C., Gustafson, E.H., Perez-Gonzalez, A., Delhomme, N., Ghavi-Helm, Y., Wilczyński, B., Riddell, A., and Furlong, E.E.M. (2012). Tissue-specific analysis of chromatin state identifies temporal signatures of enhancer activity during embryonic development. Nat. Genet. *44*, 148–156.
44. Lu, B., Zeng, Z., and Shi, T. (2013). Comparative study of de novo assembly and genome-guided assembly strategies for transcriptome reconstruction based on RNA-seq. Sci. China Life Sci. *56*, 143–155.
45. Delás, M.J., Sabin, L.R., Dolzhenko, E., Knott, S.R., Munera Maravilla, E., Jackson, B.T., Wild, S.A., Kovacevic, T., Stork, E.M., Zhou, M., et al. (2017). lncRNA requirements for mouse acute myeloid leukemia and normal differentiation. eLife *6*, e25607.
46. Liu, S.J., Nowakowski, T.J., Pollen, A.A., Lui, J.H., Horlbeck, M.A., Attenello, F.J., He, D., Weissman, J.S., Kriegstein, A.R., Diaz, A.A., and Lim, D.A. (2016). Single-cell analysis of long non-coding RNAs in the developing human neocortex. Genome Biol. *17*, 67.
47. Mirsafian, H., Manda, S.S., Mitchell, C.J., Sreenivasamurthy, S., Ripen, A.M., Mohamad, S.B., Merican, A.F., and Pandey, A. (2016). Long non-coding RNA expression in primary human monocytes. Genomics *108*, 37–45.
48. Wang, L., Park, H.J., Dasari, S., Wang, S., Kocher, J.P., and Li, W. (2013). CPAT: coding-potential assessment tool using an alignment-free logistic regression model. Nucleic Acids Res. *41*, e74.
49. Lin, M.F., Jungreis, I., and Kellis, M. (2011). PhyloCSF: a comparative genomics method to distinguish protein coding and non-coding regions. Bioinformatics *27*, i275–i282.
50. Plaza, S., Menschaert, G., and Payre, F. (2017). In search of lost small peptides. Annu. Rev. Cell Dev. Biol. *33*, 391–416.
51. Heyn, P., Kircher, M., Dahl, A., Kelso, J., Tomancak, P., Kalinka, A.T., and Neugebauer, K.M. (2014). The earliest transcribed zygotic genes are short, newly evolved, and different across species. Cell Rep. *6*, 285–292.
52. Artieri, C.G., and Fraser, H.B. (2014). Transcript length mediates developmental timing of gene expression across Drosophila. Mol. Biol. Evol. *31*, 2879–2889.
53. Wilk, R., Hu, J., Blotsky, D., and Krause, H.M. (2016). Diverse and pervasive subcellular distributions for both coding and long noncoding RNAs. Genes Dev. *30*, 594–609.
54. Gratz, S.J., Ukken, F.P., Rubinstein, C.D., Thiede, G., Donohue, L.K., Cummings, A.M., and O'Connor-Giles, K.M. (2014). Highly specific and efficient CRISPR/Cas9-catalyzed homology-directed repair in Drosophila. Genetics *196*, 961–971.
55. Quintero-Cadena, P., and Sternberg, P.W. (2016). Enhancer sharing promotes neighborhoods of transcriptional regulation across eukaryotes. G3 (Bethesda) *6*, 4167–4174.
56. Sigova, A.A., Mullen, A.C., Molinie, B., Gupta, S., Orlando, D.A., Guenther, M.G., Almada, A.E., Lin, C., Sharp, P.A., Giallourakis, C.C., and Young, R.A. (2013). Divergent transcription of long noncoding RNA/mRNA gene pairs in embryonic stem cells. Proc. Natl. Acad. Sci. USA *110*, 2876–2881.
57. Core, L.J., Waterfall, J.J., Gilchrist, D.A., Fargo, D.C., Kwak, H., Adelman, K., and Lis, J.T. (2012). Defining the status of RNA polymerase at promoters. Cell Rep. *2*, 1025–1035.
58. Reim, I., and Frasch, M. (2005). The Dorsocross T-box genes are key components of the regulatory network controlling early cardiogenesis in Drosophila. Development *132*, 4911–4925.
59. Hamaguchi, T., Yabe, S., Uchiyama, H., and Murakami, R. (2004). Drosophila Tbx6-related gene, Dorsocross, mediates high levels of Dpp and Scw signal required for the development of amnioserosa and wing disc primordium. Dev. Biol. *265*, 355–368.
60. Reim, I., Lee, H.H., and Frasch, M. (2003). The T-box-encoding Dorsocross genes function in amnioserosa development and the patterning of the dorsolateral germ band downstream of Dpp. Development *130*, 3187–3204.
61. Lagha, M., Bothma, J.P., and Levine, M. (2012). Mechanisms of transcriptional precision in animal development. Trends Genet. *28*, 409–416.
62. Mackay, T.F., Richards, S., Stone, E.A., Barbadilla, A., Ayroles, J.F., Zhu, D., Casillas, S., Han, Y., Magwire, M.M., Cridland, J.M., et al. (2012). The Drosophila melanogaster genetic reference panel. Nature *482*, 173–178.
63. Kaessmann, H. (2010). Origins, evolution, and phenotypic impact of new genes. Genome Res. *20*, 1313–1326.
64. Kondo, S., Vedanayagam, J., Mohammed, J., Eizadshenass, S., Kan, L., Pang, N., Aradhya, R., Siepel, A., Steinhauer, J., and Lai, E.C. (2017). New genes often acquire male-specific functions but rarely become essential in *Drosophila*. Genes Dev. *31*, 1841–1846.
65. Chen, S., Zhang, Y.E., and Long, M. (2010). New genes in Drosophila quickly become essential. Science *330*, 1682–1685.
66. Rossi, A., Kontarakis, Z., Gerri, C., Nolte, H., Hölper, S., Krüger, M., and Stainier, D.Y. (2015). Genetic compensation induced by deleterious mutations but not gene knockdowns. Nature *524*, 230–233.
67. Mercer, T.R., Dinger, M.E., Sunkin, S.M., Mehler, M.F., and Mattick, J.S. (2008). Specific expression of long noncoding RNAs in the mouse brain. Proc. Natl. Acad. Sci. USA *105*, 716–721.
68. Dobin, A., Davis, C.A., Schlesinger, F., Drenkow, J., Zaleski, C., Jha, S., Batut, P., Chaisson, M., and Gingeras, T.R. (2013). STAR: ultrafast universal RNA-seq aligner. Bioinformatics *29*, 15–21.
69. Haas, B.J., Papanicolaou, A., Yassour, M., Grabherr, M., Blood, P.D., Bowden, J., Couger, M.B., Eccles, D., Li, B., Lieber, M., et al. (2013). De novo transcript sequence reconstruction from RNA-seq using the Trinity platform for reference generation and analysis. Nat. Protoc. *8*, 1494–1512.
70. Wu, T.D., and Watanabe, C.K. (2005). GMAP: a genomic mapping and alignment program for mRNA and EST sequences. Bioinformatics *21*, 1859–1875.
71. Trapnell, C., Williams, B.A., Pertea, G., Mortazavi, A., Kwan, G., van Baren, M.J., Salzberg, S.L., Wold, B.J., and Pachter, L. (2010). Transcript assembly and quantification by RNA-seq reveals unannotated transcripts and isoform switching during cell differentiation. Nat. Biotechnol. *28*, 511–515.
72. Love, M.I., Huber, W., and Anders, S. (2014). Moderated estimation of fold change and dispersion for RNA-seq data with DESeq2. Genome Biol. *15*, 550.
73. Khodor, Y.L., Rodriguez, J., Abruzzi, K.C., Tang, C.H.A., Marr, M.T., 2nd, and Rosbash, M. (2011). Nascent-seq indicates widespread cotranscriptional pre-mRNA splicing in Drosophila. Genes Dev. *25*, 2502–2512.
74. Schor, I.E., Degner, J.F., Harnett, D., Cannavò, E., Casale, F.P., Shim, H., Garfield, D.A., Birney, E., Stephens, M., Stegle, O., and Furlong, E.E. (2017). Promoter shape varies across populations and affects promoter evolution and expression noise. Nat. Genet. *49*, 550–558.
75. Arvey, A., Hermann, A., Hsia, C.C., Ie, E., Freund, Y., and McGinnis, W. (2010). Minimizing off-target signals in RNA fluorescent in situ hybridization. Nucleic Acids Res. *38*, e115.
76. Cannavò, E., Koelling, N., Harnett, D., Garfield, D., Casale, F.P., Ciglar, L., Gustafson, H.E., Viales, R.R., Marco-Ferreres, R., Degner, J.F., et al. (2017). Genetic variants regulating expression levels and isoform diversity during embryogenesis. Nature *541*, 402–406.

77. Davis, M.P., Carrieri, C., Saini, H.K., van Dongen, S., Leonardi, T., Bussotti, G., Monahan, J.M., Auchynnikava, T., Bitetti, A., Rappsilber, J., et al. (2017). Transposon-driven transcription is a conserved feature of vertebrate spermatogenesis and transcript evolution. EMBO Rep. *18*, 1231–1247.

78. Pauli, A., Valen, E., Lin, M.F., Garber, M., Vastenhouw, N.L., Levin, J.Z., Fan, L., Sandelin, A., Rinn, J.L., Regev, A., and Schier, A.F. (2012). Systematic identification of long noncoding RNAs expressed during zebrafish embryogenesis. Genome Res. *22*, 577–591.

79. Zinzen, R.P., Girardot, C., Gagneur, J., Braun, M., and Furlong, E.E.M. (2009). Combinatorial binding predicts spatio-temporal cis-regulatory activity. Nature *462*, 65–70.

80. Junion, G., Spivakov, M., Girardot, C., Braun, M., Gustafson, E.H., Birney, E., and Furlong, E.E. (2012). A transcription factor collective defines cardiac cell fate and reflects lineage history. Cell *148*, 473–486.

81. Gallo, S.M., Gerrard, D.T., Miner, D., Simich, M., Des Soye, B., Bergman, C.M., and Halfon, M.S. (2011). REDfly v3.0: toward a comprehensive database of transcriptional regulatory elements in Drosophila. Nucleic Acids Res. *39*, D118–D123.

82. Kvon, E.Z., Kazmar, T., Stampfel, G., Yáñez-Cuna, J.O., Pagani, M., Schernhuber, K., Dickson, B.J., and Stark, A. (2014). Genome-scale functional characterization of Drosophila developmental enhancers in vivo. Nature *512*, 91–95.

STAR★METHODS

KEY RESOURCES TABLE

REAGENT or RESOURCE	SOURCE	IDENTIFIER
Antibodies		
Mouse monoclonal anti-Futsch (clone 22C10)	DSHB (University of Iowa)	RRID: AB_528403
Anti-Digoxigenin-POD, Fab fragments	Roche	Cat. 11207733910; RRID: AB_514500
Anti-Fluorescein-POD	Roche	Cat. 11426320001; RRID: AB_514503
Biotin Monoclonal Antibody (clone Z021) HRP	Thermo Fisher Scientific	Cat. 03-3720; RRID: AB_2522266
Chemicals, Peptides, and Recombinant Proteins		
T7 RNA polymerase	Roche	10881767001
SP6 RNA polymerase	Roche	10810274001
DIG labeling mix	Roche	11277073910
Biotin RNA Labeling Mix	Roche	11685597910
Fluorescein RNA Labeling Mix	Roche	11685619910
Western Blocking Reagent Solution	Roche	11921673001
Critical Commercial Assays		
NEBNext Ultra Directional RNA Library Prep Kit for Illumina	NEB	E7420
TSA-Plus Cy3 and Fluor	Perkin Elmer	NEL753001KT
Deposited Data		
Raw sequencing data twi::EGFP-CBP20 line (RNaseq)	This paper	ArrayExpress: E-MTAB-4069
Raw sequencing data twi::EGFP-CBP20 line (CAGE)	This paper	ArrayExpress: E-MTAB-4070
Raw sequencing data vas::Cas9 and KO lines (RNaseq)	This paper	ENA: ERP110650
Drosophila melanogaster reference genome, R5/dm3	Berkeley Drosophila Genome Project	http://www.fruitfly.org/sequence/release5genomic.shtml
Drosophila melanogaster genome annotation, R5.55	FlyBase	ftp://ftp.flybase.net/genomes/Drosophila_melanogaster/
Genomic coordinates of novel lncRNA genes	This paper	http://furlonglab.embl.de/data/
Experimental Models: Organisms/Strains		
D. melanogaster: twi::EGFP-CBP20 line: w[1118];;P{twi::ECFP-CBP20,w[+mC] }	This paper	N/A
D. melanogaster: vas::Cas9 line: w[1118]; PBac{y[+mDint2] = vas-Cas9}VK00027	Bloomington Drosophila Stock Center	BDSC: 51324
Oligonucleotides		
Primers for FISH probe amplification by PCR	See Table S6	N/A
Primers for qPCR	See Table S6	N/A
Software and Algorithms		
STAR v2.3.1z	[68]	https://github.com/alexdobin/STAR/releases
Trinity release 2014-07-17	[69]	https://github.com/trinityrnaseq/trinityrnaseq/releases
GMAP version 2013-03-31	[70]	http://research-pub.gene.com/gmap/
Cufflinks v2.1.1	[71]	https://github.com/cole-trapnell-lab/cufflinks
DEseq2 v1.2.10	[72]	https://bioconductor.org/packages/release/bioc/html/DESeq2.html

CONTACT FOR REAGENT AND RESOURCE SHARING

Further information and requests for resources and reagents should be directed to and will be fulfilled by the Lead Contact, Eileen E.M. Furlong (furlong@embl.de).

EXPERIMENTAL MODEL AND SUBJECT DETAILS

Fly line for isolation of mesodermal cells via FACS

We used a twi::EGFP-CBP20 line harboring a fusion between the coding sequence of the EGFP and the *Drosophila melanogaster Cbp20* (*FBgn0022943*) gene, under the direct control of an early mesodermal enhancer from the *twist* gene [43], in homozygocity on chromosome.

Generation of lncRNA deletion using CRISPR/Cas9

We used a reported homology-directed replacement method [54], including vectors and protocols, to perform CRISPR/Cas9-assisted deletion of selected regions. We used flyCRISPR Optimal Target Finder (http://flycrispr.molbio.wisc.edu/) to design guide RNAs. Only guides without off targets were selected. Guides were obtained as phosphorylated oligos (from Eurofins), annealed at 95°C for 5 min, then ramped to 25°C at a rate of −0.1°C/sec and inserted into pU6-BbsI-chiRNA via BbsI restriction site. Homology arms were cloned into pHD-DsRed-attP, where for insertion of homology arm 1 AarI, and homology arm 2 SapI restriction site was used. pHD-DsRed-attP [250ng/μl] and each guide in pU6-BbsI-chiRNA [50ng/μl] in total 20 ul injection buffer were injected into *vas::Cas9* flies (Bloomington ID code 51324): $W[118];\ +;(PBac\{y[+mDint2=vas-Cas9]VK00027\}/TM3Sb);\ +$.

Hatched flies were crossed 1 on 1 with $y[1]\ w[118];\ +;\ +;\ +$ and progeny was screened for DsRed positive flies. Those showing DsRed fluorescence were further crossed with balancer chromosome flies, either on the second or the third chromosome, depending where deletion was. Final stock was made by excision of DsRed marker by crossing the deletion lines with flies expression the Cre recombinase, with genotype: $y[1]w[67c23]P\{Crey\}1b;\ +;(D^*/TM3,Sb[1]);\ +$.

Region flanking the deleted fragment was amplified by PCR and sequenced for verification.

Strategy for collection of knockout embryos for RNA-Seq

Collections of live knockout embryos carrying deletion for *XLOC_004366*, *XLOC_012225*, or *XLOC_012319*, as well as the control line *vas::Cas9* were made at 6-8h after egg laying (AEL).

Embryos were collected from heterozygous adult flies containing the lncRNA deletion in *trans* to a balancer chromosome harboring GFP under the control of an early enhancer. The following genotypes of the heterozygous stocks were used:

$$+;\ \frac{XLOC\_004366}{CyO,\ twi-Gal4,UAS-GFP};\ +$$

$$+;+;\ \frac{XLOC\_012225}{TM3,Sb,Ser,twi-Gal4,UAS-GFP}$$

$$+;+;\ \frac{XLOC\_012319}{TM3,Sb,Ser,twi-Gal4,UAS-GFP}$$

$$+;+;\ \frac{vasCas9:GFP}{TM3,Sb,Ser,twi-Gal4,UAS-GFP}$$

METHOD DETAILS

Sample collection, fluorescence activated cell sorting and RNA isolation

Collections of live *twi::EGFP* embryos were made at 3–4 hr, 4–6 hr, and 6–8 hr after egg laying (AEL). Embryos were washed with water and dechorionated for 3 min in 50% commercial bleach at room temperature. Subsequently they were washed with water and PBT, dried with blotting paper and weighted. 0.5g of embryos was transferred to a tube containing 4ml freshly prepared ice-cold Schneider's *Drosophila* Medium (Termo Fisher Scientific) without serum and with 1 μg/ml Actinomycin D (Sigma). Embryos were gently resuspended by using a P1000 with a cut tip. All subsequent steps were done at 4°C. 500 μL of embryo suspension was added to 6.5ml Schneider's media with Actinomycin D in a 15ml dounce homogenizer (Wheaton Scientific) on ice and dounced with a loose pestle 7 times. Douncing step was repeated in total of 8 times until 0.5g of embryos was processed. Material from two rounds of douncing was combined in one 15 mL tube and centrifuged at 600 rpm for 5 min at 4°C. Supernatant was transferred into a clean tube and centrifuged at 1700 rpm for 10 min at 4°C. Supernatant was discarded, and 250 μL of Schneider's media complemented with 8% fetal bovine serum and 1 μg/ml of Actinomycin D was added to the pellet. All resuspended pellets were combined into a single tube, cells were gently passed through an 18-gauge needle 5 times and sieved through a 40 μm cell strainer (BD Falcon) into a 50ml tube. Approximately 5% of the total sample was transferred into a RNase free microfuge tube and centrifuged at 800xg for

10 min at 4°C. 800 µL TRIzol (Thermo Fisher Scientific) was added to the pellet and saved for RNA isolation as an unsorted sample. The remaining sample was used for fluorescence activated cell sorting (FACS).

Cellular suspensions were run on a MoFLO cell sorter (Beckman Coulter), which was precooled and kept at 4°C during the whole procedure. The sorter was run with a 70 µm nozzle at a rate of 5,000 –10,000 cells per second. A small aliquot was re-sorted to assess purity. Only samples with > 95% GFP+ cells were kept. Sorting was performed by the EMBL Flow Cytometry Core Facility.

Sorted cells were collected in 5ml round bottom polypropylene tubes (Termo Fisher Scientifc, 05-562-10B) in 500 µL Seecof saline (6mM Na_2HPO_4, 3.67mM KH_2PO_4, 106mM NaCl, 26.8mM KCl, 6.4mM $MgCl_2$, 2.25mM $CaCl_2$, pH 6.8) supplemented with 0.1U/µl RNase inhibitor (Invitrogen). Sorted cells were aliquoted in low binding RNase free tubes and centrifuged at 800 g 10 min at 4°C. Pellet was resuspended in 200 µL TRIzol LS (Thermo Fisher Scientific) and all aliquots were pooled into a single tube before proceeding with RNA isolation.

RNA isolation was performed according to manufacturer's instructions, including an overnight precipitation step with 1 µL of 10mg/ml glycogen at -80^{0}C. RNA was treated with RNase-free DNase I (Roche) in a 50 µl-volume for 30 min and purified a second time with Agencourt RNAclean XP beads (Beckam Coulter) according to manufacturer's instructions. Samples were then resuspended in RNase-free H_2O (Ambion) and an aliquot saved for integrity analysis (see later).

Homozygous lncRNA mutant embryos were manually isolated on ice in PBT under 20x magnification, based on their absence of GFP. Only embryos at the appropriate stage were collected. After screening, embryos were gently washed and flash frozen in liquid nitrogen. RNA isolation was done as described above.

Assessment of mesodermal enrichment after FACS

We examined sample purity at different levels. First, a small portion of the isolated mesodermal cells was re-sorted to ensure that the percentage of GFP^+ events in all samples that were used in further RNA-seq library preparation was always > 95%. Mesodermal enrichment was tested at the RNA level by qRT-PCR. We compared relative levels of the mesodermal genes *tinman* (*tin*) and *twist* (*twi*) to predominately ectodermal gene *short gastrulation* (*sog*) in both sorted mesodermal cells and WE samples. On average, we observed 8 to 10-fold enrichment of the relative levels of mesodermal genes in our sorted cells, compared to the WE samples.

Preparation of nuclear extracts from sorted live cells

Sorted cells were centrifuged at 800xg for 10 min at 4°C. Supernatant was removed, and the pellet resuspended and incubated for 3 min on ice in 1ml of buffer A (15mM Tris pH8, 15mM NaCl, 60mM KCl, 1mM EDTA, 0.5mM EGTA, 0.5mM DTT, 0.34M sucrose) with 20 U of SUPERaseIn (Ambion). NP40 was added to a final concentration of 0.025% and samples were incubated for 5 min on ice. The samples were centrifuged at 1000xg for 7 min at 4°C, and the pellet washed once with buffer A. Finally, the pellet was resuspended in 400 µL Trizol and RNA isolated as noted above. Nuclear enrichment was first assessed by western blotting, probing for the enrichment of tubulin and H3 in nuclear and whole cell fractions. In addition, qPCR measuring the relative expression of nuclear genes *rox2* and *or-aca* against the control *rpl32* was performed in nuclear, mesodermal and WE fractions. On average we observed a 4-fold enrichment of tested genes in the nuclear fractions compared to mesoderm or WE samples.

Depletion of rRNA for total RNA-seq

To generate rRNA-depleted RNA-seq libraries, 2.5-5 µg of total RNA from sorted cells was reverse-transcribed using a mix of biotinylated antisense oligos [73] with PrimeScript Reverse transcriptase (Takara). The resulting RNA:DNA hybrid was subjected to pull-down using two aliquots of 100 µL strepatavidin magnetic beads (Dynabeads MyOne Streptavidin C1, Invitrogen). rRNA depleted RNA was purified with Agencourt RNAclean XP beads. Reverse transcription and streptavidin pull-down with two 100 µL aliquots of magnetic beads was repeated for the second time. Double rRNA depleted RNA was purified with Agencourt RNA clean XP beads and stored at -80^{0}C. For nuclear RNA samples the same procedure was applied, except that only a single rRNA removal was performed on the total RNA obtained (typically ~1 µg). Ribodepletion was assessed both by Bioanalyzer analysis and by qPCR.

RNA-seq library preparation and sequencing

The quality of RNA, and the extent of rRNA depletion, were assessed by running total and ribodepleted RNA on a 2100 Bioanalyzer system (Agilent) using the RNA pico kit. 10-30ng of ribodepleted high quality RNA was used for RNA-Seq library preparation with NEBNext Ultra Directional RNA Library Prep Kit for Illumina (NEB) according to manufacturer's instructions, except that a custom set of Y-shape adapters were used, harboring 6nt-long barcodes for sample multiplexing. PCR was performed with a universal primer pair (PE1.0 and PE2.0 primers from Illumina) for 14-15 cycles.

After library preparation, typically 4 libraries were multiplexed together. Equal molar amounts of each library were added to a single 0.5ml low-binding tube (Eppendorf) and the final volume was subjected to a purification/size-selection procedure using 1.4x AMPure XP beads (Beckam Coulter), to eliminate residual adaptor-dimer. Both individual and pooled libraries were assessed on a 2100 Bioanalyzer system using the DNA HS kit. Two biological replicates for each condition (three for the 6-8h unsorted) were sequenced on either a Illumina HiSeq 2000 or 2500 sequencer, using 100-bp paired-end reads. All sequencing was performed by the EMBL Genomics Core Facility. The number of mapped reads per sample (per replicate), is provided in Table S1.

5′ CAGE library preparation

We prepared 5′ CAGE libraries of one sample per condition (but not from the nuclear RNA samples). In order to have extended sequencing depth and to assess technical and biological variability associated with TSS mapping by CAGE, we also prepared four extra libraries from 6-8h mesodermal samples, corresponding to two independent biological replicates, each in two technical replicates. We followed the procedure described in Schor et al. [74], starting from 2.5 μg total RNA, except from the 3-4h samples where ~1 μg was used. Libraries were multiplexed by 4 or 10 samples, amplified for 11-15 cycles and purified as described above. An extra size-selection procedure using 1.4x AMPure XP beads (Beckam Coulter) was used at the end. Pooled libraries were assessed on a 2100 Bioanalyzer system using the DNA HS kit. Libraries were sequenced in either an Illumina HiSeq 2000 or 2500 sequencer, using 50-bp single-end reads. The number of reads per sample (per replicate), is provided in Table S1.

Double fluorescent *in situ* hybridization

We analyzed the spatiotemporal expression patterns of selected lncRNA using fluorescent *in situ* hybridization (FISH) with haptenylated probes. We selected regions common to all transcript of a gene, trying to avoid small repeated regions as described by [75]. To prepare probes specific for lncRNAs, embryonic cDNA was used as a template. Fragments were amplified by PCR using the primer sets reported in Table S6.

The amplified fragments were cloned into pCRII-TOPO or pGEM-T Easy and used as templates for *in vitro* transcription, after plasmid linearization with a restriction enzyme with a unique cutting site at the opposite end of the cloned region. For *XLOC_012225*, both fragments where pooled and use together. Probes for *AbdB*, *Antp*, *Doc1*, *ftz*, *GATAe* and *sim* were prepared from cDNAs on the DGCr1 and 2 collections (http://www.fruitfly.org/DGC/).

Digoxigenin-, biotin- or FITC-labeled RNA probes were prepared using RNA labeling mixture (Roche) and T3, T7 or SP6 RNA polymerase (Roche) according to the manufacturer's instructions. After RNA synthesis, template DNA was degraded using 2 μL of RNase-free DNase I (Roche). Probes were not carbonated. RNA was precipitated at −20°C overnight by adding 1/10 volumes of 3M NaAc pH 5.2, 1/5 volumes of 6M LiCl, 200 μg tRNA as carrier and 5 volumes of absolute ethanol. After washing with 70% ethanol, pellets were resuspended in 100 μL of Hyb-A buffer (50% formamide, 5x SSC, 100 μg/ml salmon sperm, 0.1% Tween-20) by incubation for 10' at 37°C and pipetting.

Fixed dechorionated embryos (20' with 4% formaldehyde, typically stored in methanol or ethanol at −20°C), were transferred to a 1.5- or 2 mL microfuge tube, washed in 1ml PBT (PBS with 0.1% Tween-20) with decreasing proportions of methanol (70%, 50% and 30%) for 5′ each time at room-temperature, and then twice in PBT alone. Then we performed a post-fixation step for 20' in in 4% formaldehyde in PBT. Immediately after this, embryos are washed 5 times with PBT for 5′, and then once in 1:1 PBT/Hyb-B (50% formamide, 5x SSC) and once in Hyb-B. Then the embryos were pre-hybridized in Hyb-A at 65°C for at least 3.5h, before adding the denatured (10' at 80°C followed by incubation on ice) RNA probe (or mix of probes if a double *in situ* is being performed), diluted 1:50 in Hyb-A solution. We typically pre-incubate in 250-500 μL of Hyb-A, aspirate after the incubation and then add then add the diluted probe in 250 μL of total volume. After incubation overnight at 65°C, embryos were washed 6 times with Hyb-B at the same temperature, the first 3 for 30' and the second 3 for 1h. Then we performed 15' washes at room temperature with increasing proportions of PBT (20%, 50% and 80%) and finally 4 washes with PBT alone.

The probes were sequentially detected with peroxidase-conjugated antibodies (Roche), after pre-blocking 2x for 30' in Western-blot blocking reagent (Roche) diltued 1:5 in PBT and developed using the TSA-plus Tyramide fluorescence system (Perkin Elmer). Incubations with antibody were performed overnight and 4°C and then embryos were washed 6x with PBT for 20' at room temperature before proceeding with the TSA reaction. For each additional antibody incubation (if more than one probe has to be detected), we inactivate the peroxidase from the previous antibody by incubating 5′ with 10mM HCl + 0.2% Tween-20, washing 2x for 5′ and then repeating this procedure once more. A second inactivation with 3% H_2O_2 in water for 1h was applied, followed by 6x 20' washes with PBT.

Futsch protein was detected using the 22C10 monoclonal antibody (DSHB, Antibody Registry ID: AB_528403).

Viability assays for transgenic flies

For viability assays, we used the heterozygous stocks described above for *XLOC_004366*, *XLOC_012225* or *XLOC_012319* mutants. To assess mutant viability, before setting up the cross, virgin females were fed with yeast for 24 h. After setting up a cross with heterozygous parents, one set of vials was put at 25°C and another at 29°C. Parents were removed after 24h and the progeny of these crosses developing at different temperatures was analyzed after hatching.

For starvation assay, flies aged between 4 to 5 days were anesthetized with CO_2 and placed in plastic vials containing 1% agarose dissolved in water. Vials were kept in an incubator with 12:12 LD light conditions at 25°C and controlled humidity. Flies were checked once per day. The expected Mendelian ratio of progeny genotypes was observed in all cases. In addition, there was no visible increase in the number of unfertilized eggs with respect to a standard cross.

Quantification of lncRNA expression levels by qRT-PCR

For qRT-PCR analysis, we performed RT reactions on 2 μg of the indicated RNA, using the Superscript II enzyme with random hexamers as primers (Thermo Fischer Scientific), and analyzed transcript quantities for each sample against a standard curve made with dilutions of a pool of all samples. *rpl32* mRNA, a housekeeping gene, was used to normalize between samples. Three independent collections (biological replicates) were used to compare between twi::EGFP and vas::Cas9 lines. Two independent

collections were used when comparing between the DGRP samples, which were described previously [76]. The designed primer sequences for the lncRNA detection are shown in Table S6.

QUANTIFICATION AND STATISTICAL ANALYSIS

Mapping and assembling pipeline

Reads were pre-processed with Trimmomatic version 0.30 (https://github.com/timflutre/trimmomatic) to trim the first nucleotide. Before mapping, FastQC (http://www.bioinformatics.babraham.ac.uk/projects/fastqc) was used to confirm sequence quality. To generate a comprehensive transcriptome assembly out of the six considered conditions (WE 3-4h, WE 4-6h, WE 6-8h, Meso 3-4h, Meso 4-6h, Meso 6-8h) a combination of *de novo* and reference-based assembly strategies were used. Sample reads were aligned to the *Drosophila melanogaster* genome dm3 (corresponding to BDGP5) (soft-masked using ENSEMBL release 70) with *STAR* version 2.3.1z [68]. Reads from biological replicates were aligned together. Reads from nuclear enriched samples of developmental times 3-4h and 6-8h were mapped with Meso 3-4h and Meso 6-8h samples respectively. The mapper was run with options "–alignIntronMax 100000–alignMatesGapMax 500–outFilterIntronMotifs RemoveNoncanonical–outFilterType BySJout–outSAMunmapped Within." The genome was formatted with *STAR* with option "–runMode genomeGenerate–sjdbOverhang 100" and providing the FlyBase version r5.55 annotations. The read alignments were post processed to remove soft clipped bases. Individual reference-based transcript assemblies were generated for each condition with *cufflinks* [71] version 2.1.1. The FlyBase reference annotations version r5.55 were used to guide the assemblies.

In parallel, *Trinity* [69] release 2014-07-17 was used to assemble transcript isoforms *de novo*. Assembled transcripts were projected onto the *Drosophila melanogaster* genome dm3 with the splice mapper *GMAP* [70] version 2013-03-31. The resulting *cufflinks* and *GMAP* annotations were used as input to *cuffmerge* v2.1.1 to produce the comprehensive assembly.

Filtering pipeline and differential expression analysis

The comprehensive assembly was filtered to reduce the number of genes to a high quality (HQ) smaller novel set (outlined in Figure S2A). All genes whose exons overlapped by at least one nucleotide with an annotated protein coding or non-coding exon (FlyBase r5.55) on the same orientation were discarded. To prevent the inclusion of pre-mRNAs, genes with exons fully contained in annotated introns (FlyBase r5.55) in the same orientation were also discarded unless also overlapping an annotated antisense exon.

Three additional filters were applied to the monoexonic transcripts: First, ambiguous unstranded monoexonic transcripts returned by *cufflinks* were removed. Second, the entire set of monoexonic transcripts was scanned for possible events of DNA contamination. For each sample, and each monoexonic transcript, the ratio of reads mapping to the annotated strand over the total reads mapping to the locus was measured. Monoexonic transcripts were retained if they have a ratio of 0.8 or higher in at least one sample. Third, we applied a strict filter on transcript length; monoexonic transcripts < 500 nt were discarded.

Transcripts mapping to unsorted (U), unsorted extra (Uextra) and mitochondrial genome annotations or shorter than 200nt were discarded. The remaining genes were required to have a minimum expression level of 2 reads per kilobase of transcript per million mapped reads (FPKM) in at least one condition as estimated by *cuffquant/cuffnorm* [71] version 2.2.1. These transcripts were filtered to remove entries with a high content of repeats or low complexity regions, using repeatMasker soft-masked nucleotides (as in ENSEMBL version 70). Transcripts containing low complexity or repeated regions for more than 90% of their coverage were discarded. This resulted in 689 retained transcripts (from 497 genes).

We further reduced the transcripts by removing redundant transcripts, discarding isoforms differing by just a few nucleotides. For each gene, all pairs of isoforms were compared and measured using the *jaccard* distance with *BEDtools2* (https://github.com/arq5x/bedtools2) version 2.22.1, as previously described [77]. If a pair of isoforms showed a *jaccard* distance score above 0,95 then the smallest isoform was removed. The retained transcript set (663 transcripts (from 497 genes) was also scanned to detect potential degradation leftovers of mRNA maturation. For each monoexonic transcript embedded in annotated introns in the same orientation, the expression ratio between nuclear enriched and non-nuclear enriched samples at 3-4h and 6-8h was measured. Transcripts with a log 2 ratio > 1 were discarded. For this analysis the expression was measured as *cuffquant/cuffnorm* version 2.2.1 FPKMs.

The remaining 532 transcripts (367 genes) were manually inspected to detect possible read-through events, resulting in the removal of 53 genes. To define differentially expressed genes, *HTSeq-counts* version 0.6.1 and *DESeq2* [72] version 1.2.10 were used (results shown in Figure S1). Genes were considered differentially expressed and added to the "main set" of lncRNAs if they had a Benjamini-Hochberg adjusted *P*-value < 0.01 in at least one of the following comparisons:

1) S34_VS_S46, 2) S34_VS_S68, 3) S34_VS_U34, 4) S46_VS_S68, 5) S46_VS_U46, 6) S68_VS_U68, 7) U34_VS_U46, 8) U34_VS_U68, 9) U46_VS_U68, 10) Nuclear34S_VS_Nuclear68S, 11) FacsSorted_VS_facsUnsorted, 12) Time34_VS_Time46, 13) Time34_VS_Time68, 14) Time46_VS_Time68, where S = FACS sorted, U = unsorted (WE), 34 = 3-4 hours of development, 46 = 4-6 hours of development, 68 = 6-8 hours of development, Nuclear34S = RNA isolated from nuclei extracted from sorted cells at 3-4 hours. These comparisons identified 114 differentially expressed genes (195 transcripts) in one or more condition.

The 200 genes (279 transcripts) that failed to show significant differential expression were added to the "constitutive" lncRNA group, after passing through two additional filters: First a stringent minimum FPKM filter of higher or equal to 3 reads was applied. Second, we discarded transcripts fully embedded in annotated introns on the same strand. These transcripts remained after the initial

intronic transcript filtering as they also overlap a gene in the other strand and were classified therefore as exonic-antisense and not as intronic-sense.

The main set and constitutive lncRNAs were merged to generate a high quality set (HQ) of 179 genes (307 transcripts) (Figure S2A), which were used in the remainder of this study.

Comparison with the latest genome annotation

Our study was conducted using BDGP5 coordinates and annotation. A recent update to the *Drosophila* genome annotation (BDGP6) has greatly increased the number of annotated lncRNA genes by roughly 50% (from 1602 to 2507). We searched our set of new transcripts for significant overlap with this collection of 2507 lncRNA genes in the FlyBase release 6.21. By reciprocal lifting over our novel set to BDGP6 genomic coordinates, and the FB r6.21 lncRNA set to BDGP5 coordinates, we detected only 18 genes where any overlap (> 1 base on the same strand) exists between exonic sequences of both sets. From these 18 genes, only 9 have an annotated model that matches our RNA-seq models, and are therefore currently annotated, for two of which our model potentially indicates new isoforms of these genes (Table S7). Therefore, only 5% (9/179) of the lncRNA genes discovered here correspond to currently annotated FB r6.21 transcripts.

Coding potential analysis

The coding capability of our HQ lncRNA gene set, currently annotated lncRNAs and protein coding transcripts was measured using *CPAT* [48] version 1.2.1. Following the developers' indications, we took a cut-off value of 0.39 to assign evidence of coding potential by this method (Figure 1C).

NCBI *BLASTX* and *RPSTBLASTN* (version 2.2.29+) were run with options -evalue 100 -strand plus -num_alignments 1. We assessed whether a translated product of all possible ORFs matched any annotated proteins in the *Drosophila* proteome and Swiss-Prot datasets from UniProt (https://www.uniprot.org/) release 2014_08 using *BLASTX* (Figure S2B). *RPSTBLASTN* was run against the PFAM database from the NCBI's Conserved Domain Database (CDD) FTP-archive (rev. 20 February 2014) to check whether the translated product of the transcripts matched annotated protein domains. In all cases, an E-value of 0.01 was taken as threshold.

Lastly, we performed an ORF conservation analysis by examining the evolutionary signature across 12 *Drosophila* species. We used the exonic coordinates of our HQ lncRNA gene set to extract the MAF genome alignments available from UCSC table browser (https://genome.ucsc.edu/cgi-bin/hgTables?). Galaxy (https://usegalaxy.org/) was used to process the MAF alignments by reverse complementing the blocks derived from negative strand exons, and binding the blocks corresponding to the same transcript. Next we used the seq_reformat tool part of *T-Coffee* (http://tcoffee.crg.cat/) version_11.00.8cbe486 to remove *Anopheles gambiae* and *Tribolium castaneum* species from the alignments and to rename the FASTA headers to use the same syntax adopted by *PhyloCSF* [49]. Finally we ran *PhyloCSF* with options '12flies–orf = ATGStop–frames = 3–aa–removeRefGaps'. Following previous reports [78], we used a PhyloCSF score of 20 decibans as threshold, since it allows for a good separation between known coding and non-coding transcripts.

All scores for these three tests of coding potential are included in Tables S2 (novel genes) and S3 (annotated genes). The tables also provide a classifier that identifies genes producing transcripts that have values above threshold for two of these tests (CPAT, BLAST searches and PhyloCSF).

Improving the annotation of the 5′ end of the lncRNA by TSS clipping and extension

The HQ lncRNA transcription start sites (TSSs) were scanned to identify putative *cuffmerge* assembly artifacts or alternative TSSs; weakly supported transcripts mapping at the 5′ end of genuine highly expressed transcripts can be erroneously fused by *cuffmerge*, falsely expanding the TSS toward the 5′. Similarly, alternative TSSs can blur alignments in ChIP-Seq and CAGE analyses. The following approach was applied to post-process *cuffmerge* assemblies and clip scantly supported TSS: For each HQ novel transcript, the region in the proximity of the predicted TSSs was surveyed to identify CAGE signals supportive of a high quality transcription start site. The region considered in the analysis included up to 800 nt upstream the *cuffmerge* transcript start, and up to 30% the transcript length moving from the *cuffmerge* transcript start toward the transcript center. The TSS was redefined within this region to match the closest CAGE peak of at least 50 read if there was a CAGE peak. A total of 71 transcripts exhibited affected 5′ ends, of which 24 were expanded toward the 5′, and 47 reduced in size. The mean number of clipped or expanded nucleotides is 110. The quality of the modified transcripts 5′ ends was assessed visually and by comparison to ChIP-seq (Figure 1D) and CAGE (Figure S2C) data, as described below.

Histone mark and pol II support of mesodermal promoters

RNA Polymerase II, H3K4me3, H3K4me1, H3K47ac ChIP-Seq libraries from mesodermal cells isolated from 6-8h embryos generated in our previous study [43] were used to support the promoters of HQ lncRNAs, annotated lncRNAs and protein coding transcripts. To reduce redundancy, for each gene, we considered non-overlapping TSS (within a window of 50 nt on either strand). When overlapping TSS occurred, the promoter associated with the highest expressed isoform (highest *cuffnorm* FPKM) was kept. Additionally, to reduce the confounding signal originating from the TSSs of close genes, we considered just transcripts whose TSS did not overlap any other TSS predicted in the unfiltered *cuffmerge* set in an area of ± 0.2 Kb. We focused on transcripts that are expressed in the same condition as the ChIP-Seq libraries (*cuffnorm* gene FPKM > = 1 on Meso 6–8 hr saamples). Figure 1D shows the mean support of mesodermal promoters (+/−0.2 Kb) for each library, expressed as normalized and background corrected (Input for Pol II and total H3 for histone modifications) ChIP-Seq read counts. The plots were generated using *computeMatrix* version 1.5.9.1 of the *deeptools* suite (https://deeptools.readthedocs.io/en/develop/).

5′ CAGE mapping and support of transcription start sites

CAGE reads from the 10 libraries were demultiplexed and trimmed to obtain the 27nt-tags. We also removed the first nucleotide of the tag, which is frequently an extra G added by the reverse transcriptase, as observed before [74]. This resulted in 26nt-long tags, which were mapped against the *Drosophila melanogaster* genome dm3 with *bowtie2* version 2.2.3 with option–very-sensitive. In the analysis shown in Figure S2C, mapped CAGE tags are used to support transcription start sites (TSS) (+/−0.2 Kb) of our HQ lncRNA set, currently annotated lncRNAs and protein coding transcripts. To reduce redundancy, for each gene, we considered non-overlapping TSS (within a window of 50 nt on either strand). Only genes expressed in the same condition as the considered CAGE library were included (*cuffnorm* gene FPKM > = 1). Plots were generated using *computeMatrix* version 1.5.9.1 of the *deeptools* suite (https://deeptools.readthedocs.io/en/develop/). The counts are normalized by library sizes. The 6-8h curve shows the average support across all the replicated libraries.

Ribodepleted versus Poly-A+ selected libraries:

We compared the expression of our HQ lncRNA transcripts between total ribo-depleted RNA and poly-A+ RNA-seq isolated from the same set of samples from the whole-embryo at 6-8h, therefore allowing direct comparison in matching stages and conditions. Libraries were prepared as described above, except that the double ribodepletion step was replaced by poly-A selection following the instructions from the Ultra Directional RNA Library Prep Kit for Illumina (NEB) .

The average transcript FPKM scores between replicates was used as expression values. To account for developmental time and tissue specificity in this analysis we considered just the HQ transcripts expressed in whole-embryo 6-8h samples with an FPKM of at least 2 in the ribo-depleted samples. The heatmaps in Figure 1F show the log2 transformed FPKM values estimated by *cuffquant*/*cuffnorm* (version 2.2.1) increased by a pseudo-count of 0.1.

lncRNA classification

HQ lncRNAs and annotated lncRNA genes were classified with respect to their genome location using the genome annotation (FlyBase version r5.55) (Figure 3A) - protein coding genes were considered if expressed in the experimental conditions (resulting from the filtering described in Figure S2A). The classification is mutually exclusive and based on the overlap between each gene and several features in the following rank: TSS > TES > exon > intron > promoter > enhancer > intergenic.

The promoter was defined as the area 1 kb upstream of an annotated TSS. The enhancer set is a superset containing both characterized embryonic enhancers (from transgenic assays) and putative enhancers from TF occupancy, based on the following data: 1) 8008 *cis*-regulatory modules (CRMs) defined by ChIP for multiple mesodermal transcription factors [79]; 2) ~4000 Tin bound putative cardiac CRMs [80]; 3) CRM activity database [43]; 4) RedFly version 3.3 [81]; 5) Vienna tiles enhancer dataset (except elements classified as always inactive) [82].

lncRNA-PCG expression correlation

The two sets of lncRNA (our HQ novel lncRNA set and currently annotated lncRNA transcripts) were merged to generate a comprehensive set of 588 lncRNAs expressed during these embryonic stages. To assigned each lncRNA to a protein coding gene (PCG) in its vicinity (Figure 3B), the following six mRNA-lncRNA pair sets were considered:

1. Pairs of lncRNAs and the closest annotated PCG (either overlapping or not). To generate this set, *BEDtools2 closest* v2.22.1 was used in combination with the longest isoform for each gene.
2. Pairs of lncRNAs and the closest non-overlapping annotated PCG. To generate this set, *BEDtools2 intersect* and *closest* v2.22.1 were used with the longest isoform for each gene.
3. Pairs of lncRNAs overlapping antisense the TSS of annotated PCG, and the corresponding protein coding transcript. This class of lncRNAs is defined following the same classification ranking already described. To remove redundant counting, if there are multiple TSSs embedded in one lncRNA, only the TSS corresponding to the longest mRNA is considered. Similarly if there are multiple lncRNAs embedding a single TSS, then only the longest lncRNA is considered.
4. Pairs of lncRNAs overlapping antisense exons of annotated PCG, and the corresponding protein coding transcript. This class of lncRNAs is defined following the same classification ranking already described. To remove redundant counting, if there are multiple PCG overlapping a single lncRNA, only the longest mRNA is considered. Similarly if there are multiple lncRNAs overlapping the same PCG, then only the longest lncRNA is considered.
5. Pairs of lncRNAs overlapping the promoter of annotated PCG (non protein overlapping) and the closest annotated PCG. This class of lncRNAs is defined following the same classification ranking already described. To remove redundant counting, if there are multiple PCG promoters overlapping a single lncRNA, only the longest mRNA is considered. Similarly if there are multiple lncRNAs overlapping the same PCG promoter, then only the longest lncRNA is considered.
6. Pairs of lncRNAs and randomly assigned annotated PCG. For each gene just the longest isoforms were used. One thousand random sets were generated and concatenated.

For each of these sets, the FPKM Pearson correlation was computed considering the transcript pairs of each set and an array of all experimental conditions (Meso 3-4h, Meso 4-6h, Meso 6-8h, Meso 3-4hNuc, WE 3-4h, WE 4-6h, WE 6-8h), as shown in Figure 3B.

Expression clusters

The merged set of lncRNAs (our HQ set and previously annotated lncRNA) was used to cluster lncRNAs in groups with similar expression properties across these different conditions. For each gene, only the longest isoform was considered. Initially nine k-means FPKM expression clusters were obtained. After filtering to remove outliers by discarding elements with a PCA distance from the cluster centroid above 20, and clusters with just one member, clustering was re-run with five k-means. Following, the clusters were filtered again to remove elements with a PCA distance from the cluster centroid above 1.5, and again clusters with less than two elements were removed. Finally, the expression correlation between each element and the median of the expressions in each condition was measured. Elements that did not show a correlation of > 0.905 were removed. This resulted in 5 robust clusters containing 36% (130 out of 362 genes) of the lncRNA genes (Figure 2B).

GO analysis

For each lncRNA group of interest, the set of neighboring genes was extracted (FlyBase version r5.55, see Tables S3, S4, and S5). The first gene mapping on each side of each lncRNA were considered, including possible antisense overlapping genes. Then the R library GOstats version 2.32.0 was used in combination with *Drosophila* annotations from the R library org.Dm.eg.db version 3.0.0 to compute the gene ontology (GO) term enrichment, using biological process and function trees. *P*-values were adjusted using Benjamini-Hochberg multiple testing correction.

Bidirectional transcription from PCG promoters

Considering an area of ± 1Kb around the TSS of PCGs, the divergent transcription from promoters of PCGs was measured using *computeMatrix* version 1.5.9.1 of the *deeptools* suite on expression data (mesodermal sorted, time 6-8h, non nuclear-enriched). The left panels of Figure 3C show sense and antisense read counts in bins of 10nt, downstream and upstream the TSS respectively. Each row indicates a promoter, order by the levels of upstream antisense expression. The orientation of first mate reads is reversed. The rightmost boxplot shows the expression difference between divergently transcribed PCGs ("top," first one third of the heatmap) and the PCGs with no divergent transcription ("bottom," last third of the heatmap).

Differential expression analysis of the KO lines

RNA-seq reads from homozygous mutant embryos were mapped to the reference genome (BDGP5.70) using STAR (v. 2.5.1b) with options "–alignIntronMax 100000–alignMatesGapMax 500–outFilterIntronMotifs RemoveNoncanonical–outFilterType BySJout–outSAMunmapped Within." Re-sequenced samples were merged. Duplicate reads were removed using Picard Tools MarkDuplicates (v. 1.139) followed by filtering with samtools. We then used htseq-count (v. 0.6.1p1) to assign reads to transcripts. To this end we used the merged annotation from Flybase r5.55 and our *de novo* identified lncRNA after quality filtering steps, as described above.

Differentially expressed genes were identified using DESeq2. The analysis was run testing for differential expression in samples carrying the same deletion versus all the others samples as control (e.g., *XLOC_012225* knockout (KO) versus *XLOC_012319* KO and the wild-type strain, vas::Cas9 to test for differential expression in *XLOC_012225* KO). We reasoned that the *loci* that were deleted in the CRISPR lines would not dramatically affect the transcriptome while including the information from all the samples would increase statistical power. *XLOC_004366* showed systematic biases in the preliminary analysis and therefore was excluded from this general scheme and analyzed individually against vas::Cas9 parental line. P values were adjusted for multiple testing using Benjamini-Hochberg procedure.

Genotype specific variation in lncRNA expression

We tested for robustness of expression of our newly discovered lncRNAs in a different *D. melanogaster* wild-type strain. LncRNA expression was compared between the Oregon R twi::EGFP-CBP20 line (used for the FACS sorting and lncRNA detection described above) and the vas::Cas9 line (using for CRISPR deletion) at 6 to 8 hours. vas::Cas9 reads were mapped using STAR with the same settings as stated above for the Oregon R based data. Cutfflinks (v2.1.1) was then used on the merged transcriptome annotation (Flybase v5.55 and HQ lncRNA set). We report the mean FPKM expression across all replicates, and compare the overlap of both sets (Figures 6A and 6B).

Conservation of lncRNA genes expressed at different stages

We separated the comprehensive set of lncRNA genes (novel + annotated) into three groups, according to their expression at the earliest (3-4h) and the latest (6-8h) time intervals. Those that did not change significantly between 3-4h and 6-8h (Time34_VS_Time68) were classified as "constitutive." From the significantly changing, we used the gene-level expression between WE samples at 3-4h and 6-8h to determine whether they were overexpressed "early" or "late."

We computed a conservation score for each transcript in the HQ sets (see Tables S2, S3, and S4), based on the UCSC phastConsElements15way score, using the following formula:

$$s = \frac{\sum_i (O_i \, P_i)}{L}$$

where *i* is the overlapping phastCons element, *O* is the number of overlapping nucleotides, *P* is the phastConsElements15way score for that element and *L* is the transcript length.

We compared the resulting per-transcript phastCons scores corresponding to the three groups of genes, selecting the longest transcript for each gene. Statistical significance was assessed with a two-sided Mann-Whitney test. Results were similar when selecting instead the highest expressed isoform or the most conserved. The differences between the medians of the “early” group versus the other two groups were similar when using only those lncRNA classified as “intergenic” (i.e., not overlapping PCGs), although the results were above the significance threshold 0.05 due to low number of genes on each group.

DATA AND SOFTWARE AVAILABILITY

The accession number for RNA-seq and CAGE raw data for the twi::EGFP embryos reported in this paper is ArrayExpress: E-MTAB-4069 (total and poly-A+ RNA-seq from) and E-MTAB-4070 (CAGE). The accession number for RNA-seq raw data for the CRISPR KO experiment is European Nucleotide Archive (ENA): ERP110650.

ADDITIONAL RESOURCES

BED files with BDGP5 and BDGP6 genomic coordinates of novel lncRNA can be downloaded from the Furlong Lab website: http://furlonglab.embl.de/data/

Current Biology, Volume *28*

Supplemental Information

Non-coding RNA Expression, Function, and Variation during *Drosophila* Embryogenesis

Ignacio E. Schor, Giovanni Bussotti, Matilda Maleš, Mattia Forneris, Rebecca R. Viales, Anton J. Enright, and Eileen E.M. Furlong

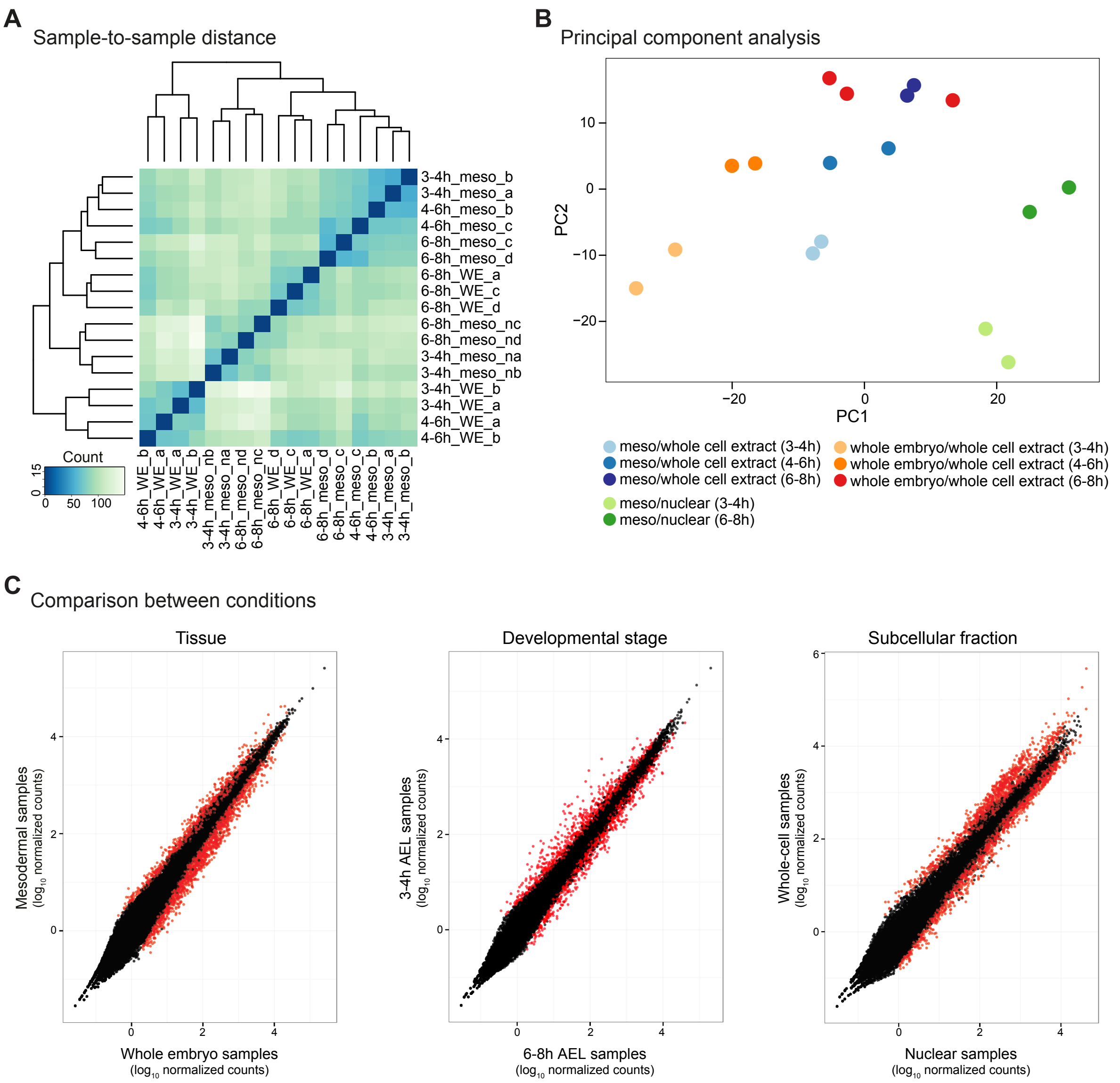

Figure S1. Differential expression analysis. Related to STAR Methods

(A) Matrix of pairwise distances between all samples included in the DE analysis. (B) Principal component analysis. S = FACS sorted (mesoderm), U= unsorted (whole embryo), yes = nuclear enrichment, no = whole cell extract. Replicates group together. Samples tend to group closer by developmental time point, then by tissue or nuclear fraction (e.g. all 3-4hr samples are in the lower half). (C) BaseMean counts (log 10 scale) as estimated by DESeq2 comparing between: *(left)* tissue (mesoderm vs whole embryo), *(middle)* developmental stage, *(right)* nuclear fraction versus whole cell. BaseMean is the mean of normalized counts of all samples of that condition, normalizing for sequencing depth. For example, the middle panel reflects gene coverage at 3-4 vs 6-8 hours, averaging all 3-4h and 6-8h samples (i.e. including both WE and mesoderm samples). The right panel reflects gene coverage in nuclear enriched vs non-nuclear enriched samples, averaging all nuclear and non-nuclear samples (which are all mesoderm samples). Scatterplots show significant genes for the indicated contrasts. Red dots depict genes with adjusted p-value < 0.01.

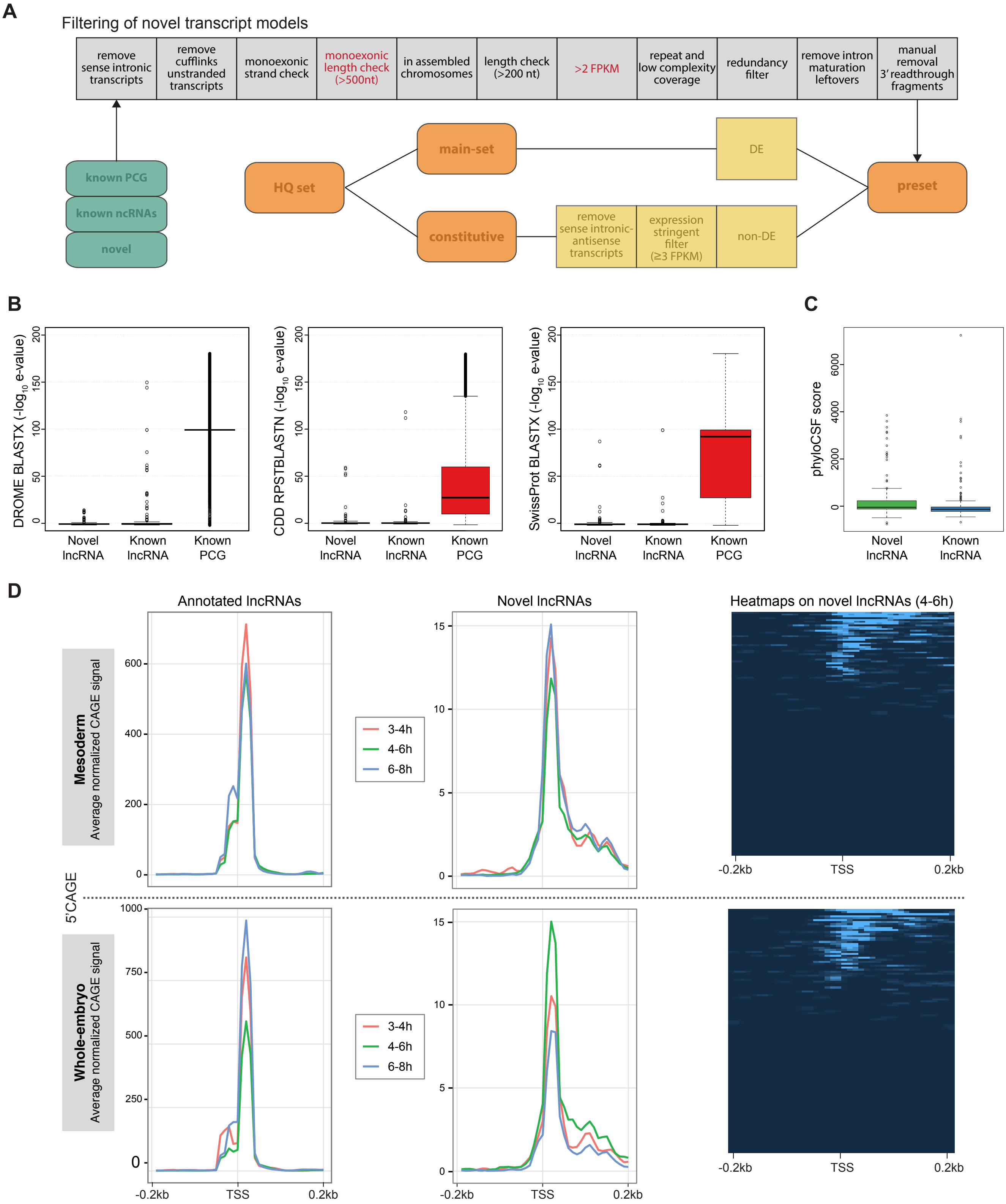

Figure S2. Identification and validation of novel lncRNA genes. Related to Figure 1
(A) Overview of transcript filtering to obtain a high-confidence set of new lncRNAs. The two most stringent filters, in terms of transcripts removed, are shown in red text. The filtered pre-set was divided into transcripts with (main-set) and without (constitutive) significant ($p < 0.01$) differential expression across stage or tissue. The combination of these two results in our high-quality (HQ) set of novel lncRNAs used in the rest of the analysis. (B) Boxplots showing the e-values of the three transcript sets (novel and known lncRNA and PCGs) for the following BLAST searches: BLASTX against Drosophila proteome (DROME) and SwissProt; RPST BLASTN against the NCBI Conserved Domain Database (CDD). (C) PhyloCSF analysis for conservation of predicted ORFs found in the novel and annotated lncRNA sets. (D) 5'CAGE support of transcript start sites. Plots show average 5'CAGE signal for promoter regions of novel and annotated lncRNAs. Transcripts with promoter region overlapping other TSSs (in a window of 50nt on the same strand) were excluded. Heatmaps represent normalized CAGE signal for novel transcripts expressed at 4-6h in mesoderm and whole embryo.

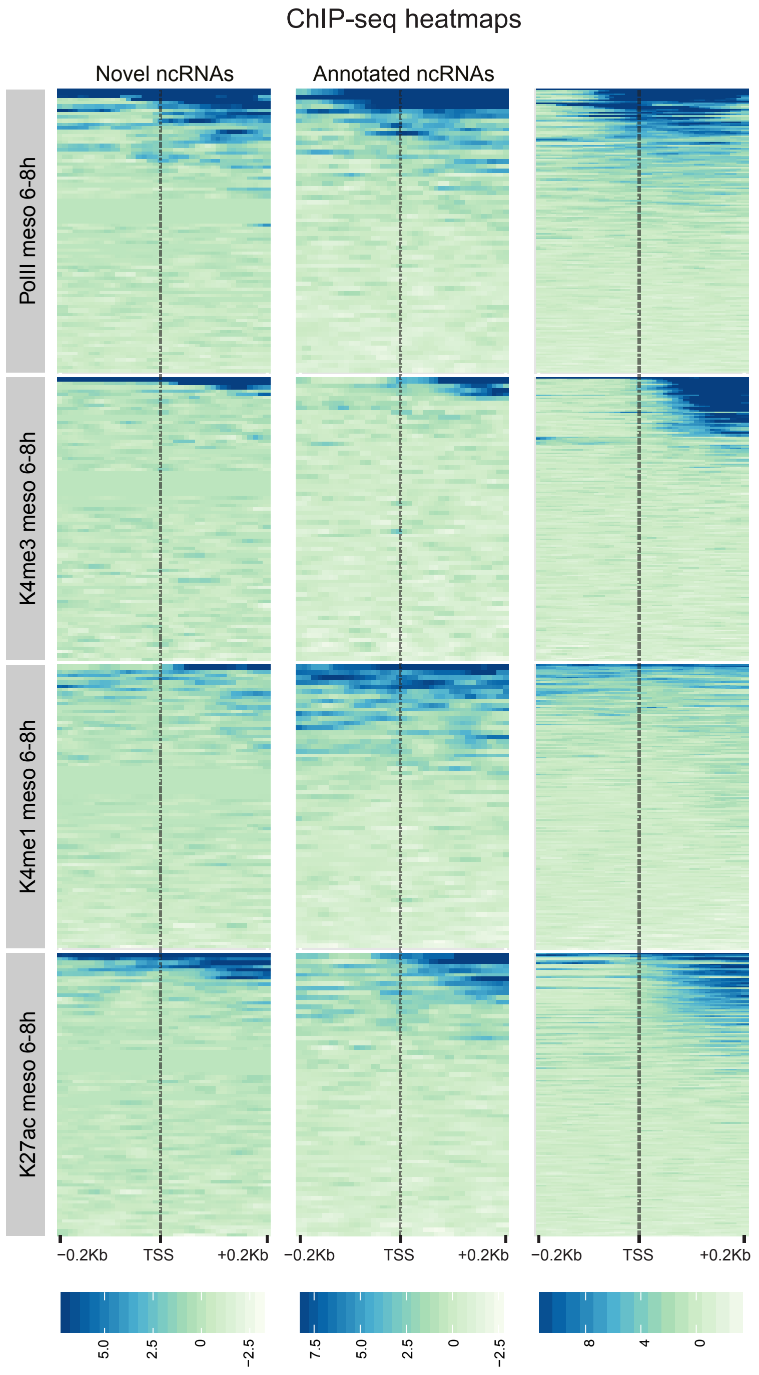

Figure S3. RNA pol II and promoter associated chromatin marks are often not detected at lncRNA promoters. Related to Figure 1

Shown are heatmaps of ChIP-seq signal for the indicated factors at novel lncRNA, annotated lncRNA and protein-coding gene promoters. Data corresponds to the average profile plots shown in Figure 1D.

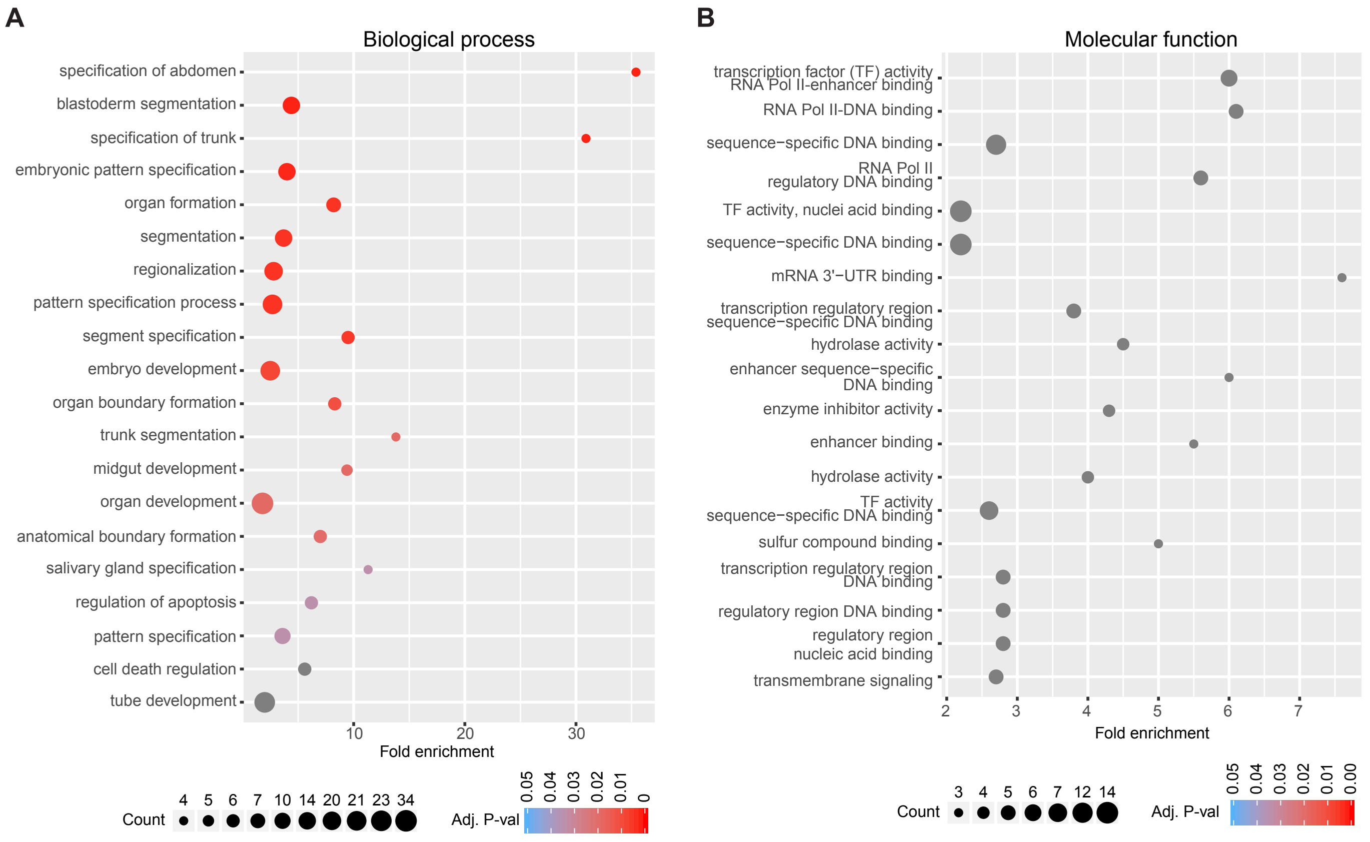

Figure S4. Functional enrichment of nuclear lncRNA-associated genes. Related to Figure 2
(A) GO term enrichment analysis of biological processes. (B) GO enrichment analysis of molecular functions. For each analysis, the first protein coding gene (PCG) neighbor at each side of the lncRNA was considered, while the entire high-quality set of PCGs expressed in our sample set was used as the reference. X-axis indicates fold enrichment between observed and expected GO terms, y-axis reports the significant biological process terms sorted by decreasing p-value. Dot size reflects the number of genes in that ontology, dot color indicates Benjamini-Hochberg adjusted p-values.

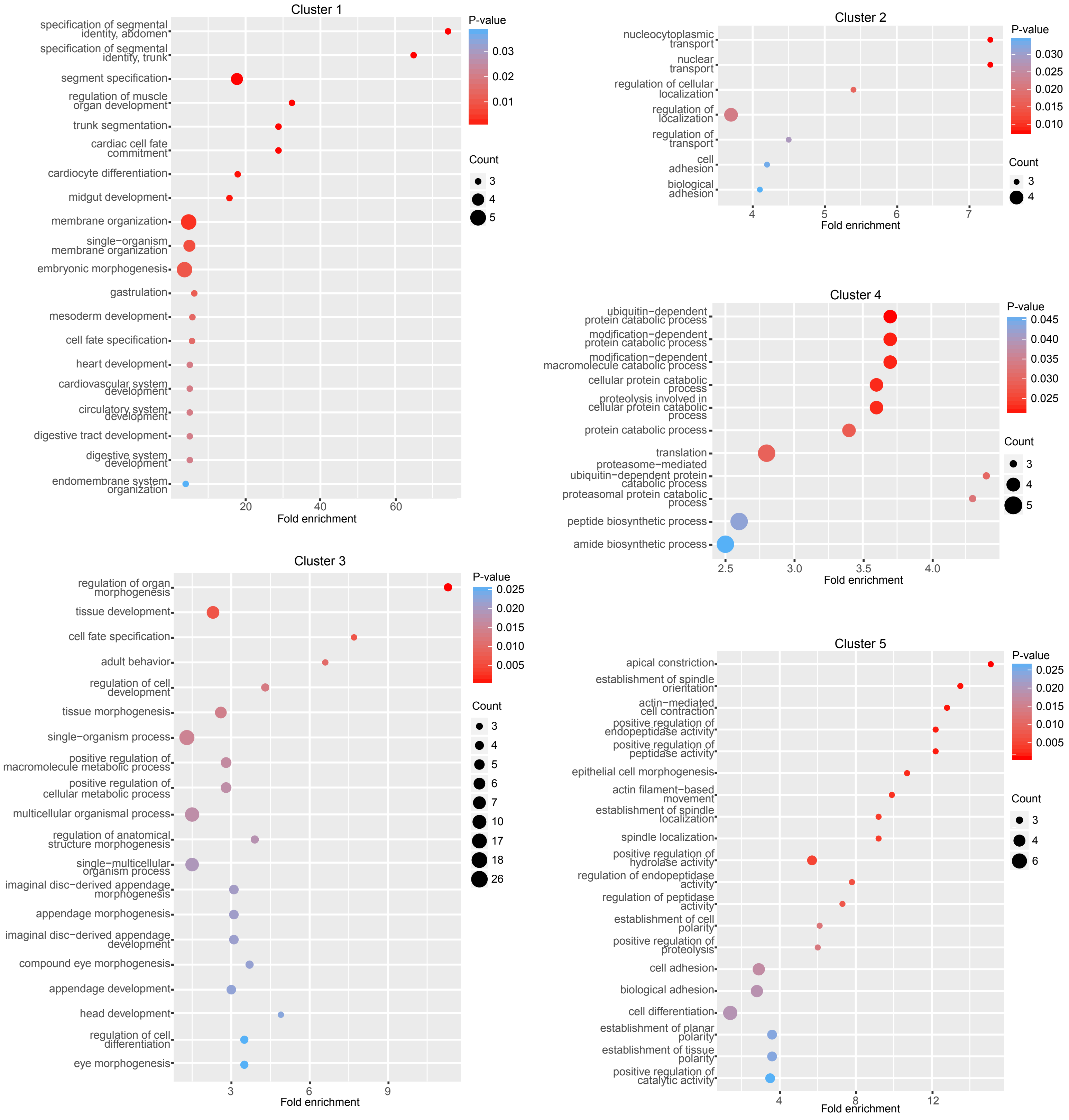

Figure S5. GO term enrichment of genes in the vicinity of lncRNA gene clusters. Related to Figure 2
Enrichment analysis of GO terms (biological processes) for the two closest protein coding gene (PCG) neighbors of lncRNA genes belonging to clusters 1 to 5. The high-quality set of PCGs expressed in our sample set was used as the reference. X-axis indicates fold enrichment between observed and expected GO terms, y-axis reports the significant biological process terms sorted by decreasing p-value. Dot size reflects the number of genes in that ontology, dot color indicates raw p-values.

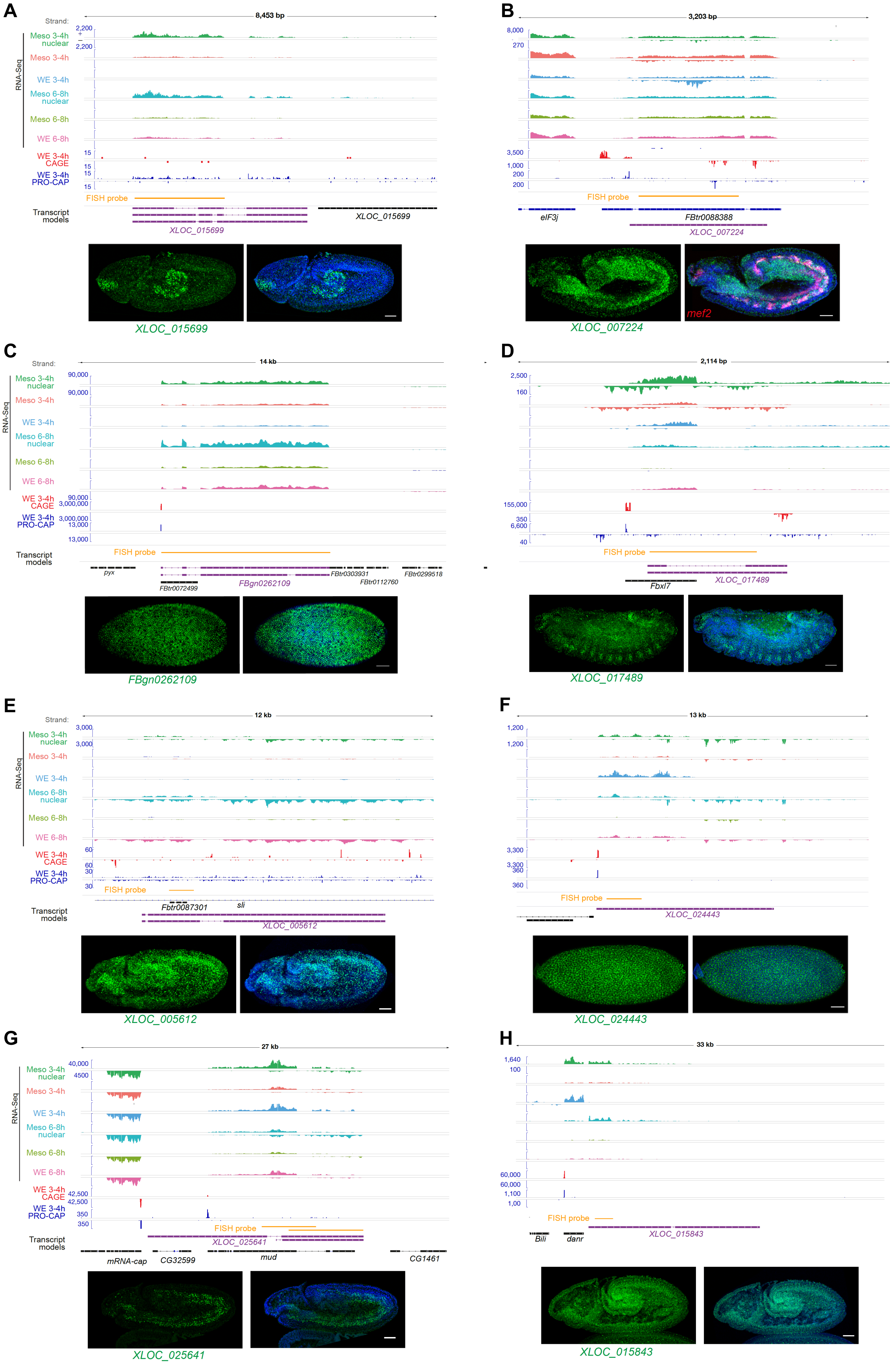

Figure S6. Temporal and Spatial expression of developmental lncRNAs. Related to Figure 2

(A-H) Above, genomic regions showing the expression of the indicated lncRNA gene (purple gene models) across samples. Meso = FACS purified mesodermal cells, WE = whole embryo, h = hours of embryogenesis. Below, fluorescent in situ hybridization (FISH) images of the lncRNA (green) with DAPI (blue) showing representative expression patterns. *XLOC_007224* is a double in-situ with a muscle marker (*Mef2*, red) (B). The coordinates and expression of all lncRNAs tested is provided in Table S5.

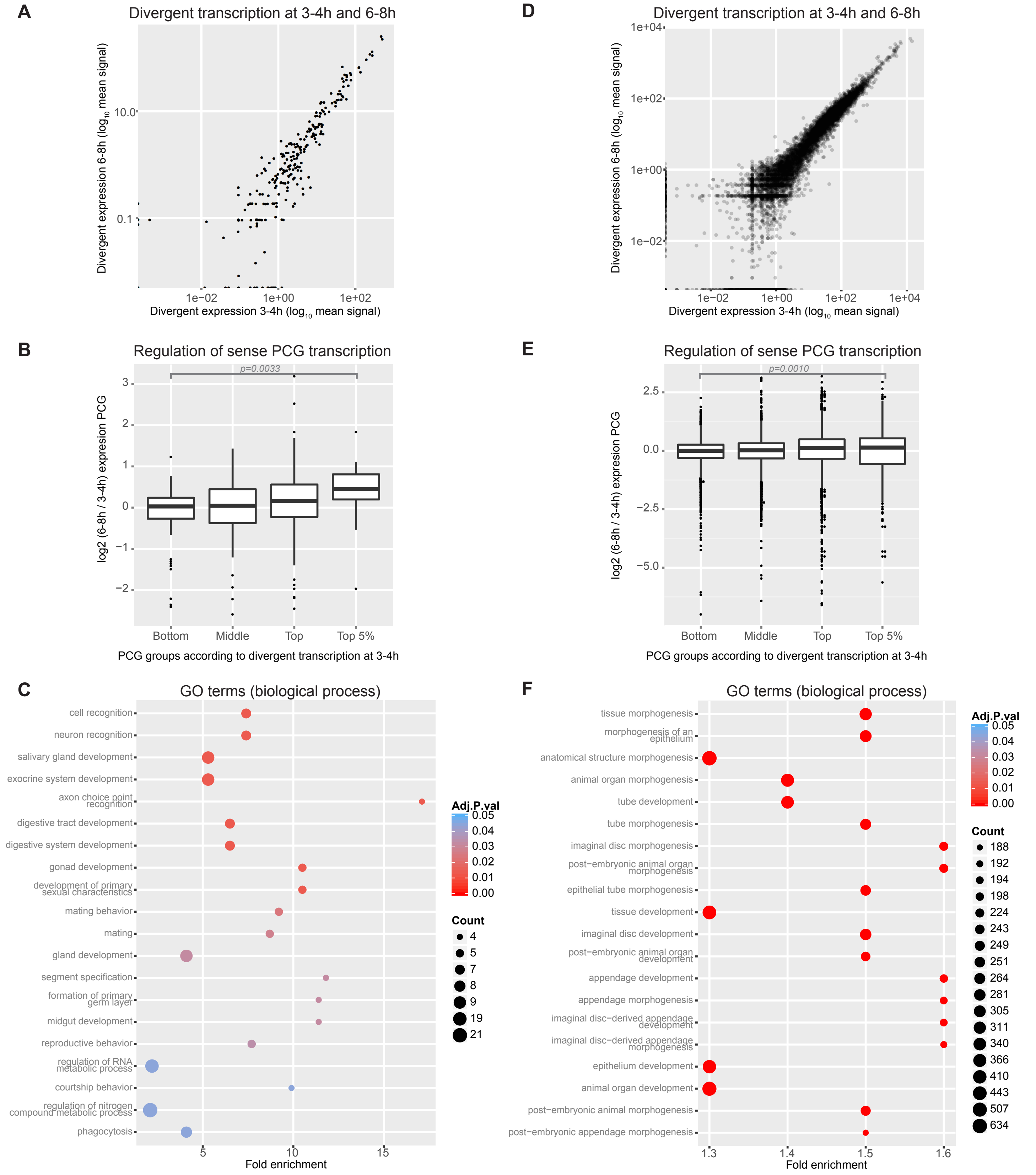

Figure S7. Analysis of divergent transcriptional units. Related to Figure 3

(A-C) lncRNA-associated PCG promoters (Figure 3C-D). (D-F) Promoters from all PCG active at mesoderm 3-4h (Figure 3E-F). (A, D) Divergent transcription is stable between 3-4h and 6-8h developmental times, as shown by the high correlation between expression values at both time-points (Pearson's r = 0.985 and 0.956 respectively). (B, E) Presence of divergent transcription predicts differential regulation of gene expression across developmental time for the PCG. Boxplots indicate change in expression from 3-4h to 6-8h of the PCGs correspondingto the different 3-4h divergent transcription groups. PCGs were divided in thirds and in addition the highest 5% is shown separately. P-value for Wilcoxon test is indicated. (C, F) Genes harboring divergent transcription (top third) are enriched in developmental functions. GO term enrichment analysis using the corresponding complete PCG set as universe (antisense lncRNA-associated PCG or all expressed in mesoderm at 3-4h.

Table S1. Overview of the transcriptome sequencing. Related to Figure 1

Summary of sequenced samples and mapped reads for the total RNA-seq and 5' CAGE analysis of gene expression on the twi::EGFP-CBP20 line.

Sample name	Origin	Library	Time point	Mapped reads
34_Sa	Mesoderm	total RNA-seq	3-4h	20121011
34_Sb	Mesoderm	total RNA-seq	3-4h	17504271
34_Sna	Mesoderm nuclei	total RNA-seq	3-4h	14580946
34_Snb	Mesoderm nuclei	total RNA-seq	3-4h	14883067
34_Ua	Whole embryo	total RNA-seq	3-4h	5055384
34_Ub	Whole embryo	total RNA-seq	3-4h	9050576
46_Sb	Mesoderm	total RNA-seq	4-6h	13402704
46_Sc	Mesoderm	total RNA-seq	4-6h	18569755
46_Ua	Whole embryo	total RNA-seq	4-6h	8769415
46_Ub	Whole embryo	total RNA-seq	4-6h	4181181
68_Sc	Mesoderm	total RNA-seq	6-8h	15781612
68_Sd	Mesoderm	total RNA-seq	6-8h	8877609
68_Snc	Mesoderm nuclei	total RNA-seq	6-8h	19991188
68_Snd	Mesoderm nuclei	total RNA-seq	6-8h	12754290
68_Ua	Whole embryo	total RNA-seq	6-8h	7058205
68_Uc	Whole embryo	total RNA-seq	6-8h	12176734
68_Ud	Whole embryo	total RNA-seq	6-8h	14744629
CAGE_U60_34h	Whole embryo	5' CAGE	3-4h	23241554
CAGE_S60_34h	Mesoderm	5' CAGE	3-4h	15876118
CAGE_U46_46h	Whole embryo	5' CAGE	4-6h	17738836
CAGE_S46_46h	Mesoderm	5' CAGE	4-6h	13564260
CAGE_U56_68h	Whole embryo	5' CAGE	6-8h	12576260
CAGE_S56_68h	Mesoderm	5' CAGE	6-8h	26555562
CAGE_meso_S13_r1	Mesoderm	5' CAGE	6-8h	24953370
CAGE_meso_S13_r2	Mesoderm	5' CAGE	6-8h	17308274
CAGE_meso_S3_r1	Mesoderm	5' CAGE	6-8h	38534497
CAGE_meso_S3_r2	Mesoderm	5' CAGE	6-8h	21603091

Table S6. Oligonucleotides used for FISH probe amplification and qPCR. Related to STAR Methods and Key Resources Table

Gene	Application	Fw Sequence (5' -> 3')	Rv Sequence (5' -> 3')
XLOC_010934	qPCR	GCCTGCAATCGTAAAGGATGG	TTTCGCACGGCTCTTGTTTC
XLOC_011009		AGCAAAAATCGCAGGCACAG	GCTGCAGCATGGAATTTTCC
XLOC_013478		TGGCAGACAACACACTTTCG	TTATTTCCCAACGGCCCTTG
FBgn0263019	cDNA amplification for FISH probe	CAAAAACGAGTCAGCGGCAA	ATGTGACTCCCGCTTTCGTT
FBgn0263595		GAAACCGAATGCGAATCCCG	ACTGGGCCATAAAGCAACCA
FBgn0266236		AGTGTCTGAATCACTGGGCG	TGGCTTTGACATTTCGTTCA
FBgn0266631		GGAAAAAGGATGCGAATCCGA	TCCTTGTTCAATCTAAGAGGCA
XLOC_004366		GGAAGGTATGGGATGGCCTG	GACGGATTTCGGAGTCGACA
XLOC_012225_1		GAATCCAAGGAGCGTGGTCA	TTGCCATTTCCATTGCAGCC
XLOC_012225_2		ATGCCCTGAAATCTTGCGGA	TAACGACGATCCAAGAGGGC
XLOC_012319		CCAGCCACGCATTTTGTCAA	TTGGCAGAGTGGGTGGTTTT
XLOC_018482		TAGAGCAGCGCGATAAAGCA	GAAGGACTTATCGGCCGTCG

Table S7. Comparison of our novel lncRNA genes set with the FlyBase r6.21 annotation. Related to STAR Methods

	Novel gene model	Transcripts in overlap	Annotated gene	Transcripts in overlap	Comments
Matching previously annotated transcripts	XLOC_004536	TCONS_00012691	CR46064	FBtr0347293	
	XLOC_005255	TCONS_00014942	CR45276	FBtr0345530	Novel model is longer
	XLOC_007166	TCONS_00020918	CR45321	FBtr0345669	
	XLOC_007956	TCONS_00023809	CR45270	FBtr0345480	Novel model is longer
		TCONS_00023810		FBtr0345792	
				FBtr0345793	
	XLOC_009721	TCONS_00028436	CR46005	FBtr0347134	Novel model is longer
		TCONS_00028437		FBtr0347135	
		TCONS_00028438			
		TCONS_00028439			
	XLOC_012319	TCONS_00036228	CR46003	FBtr0347130	
		TCONS_00036229			
		TCONS_00036230			
		TCONS_00036231			
		TCONS_00036232			
	XLOC_015145	TCONS_00043859	CR45631	FBtr0346325	
Previously annotated genes but new models may represent different transcript isoforms	XLOC_013181	TCONS_00038525	CR45912	FBtr0346984	Novel model reflects more accurately the read coverage
		TCONS_00038526		FBtr0346985	
				FBtr0346986	
	XLOC_024457	TCONS_00066961	flam	FBtr0347221	Overlap at the start region, but novel models include novel exonic region
		TCONS_00066962		FBtr0347222	
		TCONS_00066963		FBtr0347223	
		TCONS_00066964		FBtr0347224	
		TCONS_00066965		FBtr0347225	
		TCONS_00066966		FBtr0347226	
				FBtr0347227	
				FBtr0347467	
				FBtr0347468	
				FBtr0347469	
Different genes than those annotated	XLOC_000697	TCONS_00002074	CR46196	FBtr0347474	
		TCONS_00002075			
	XLOC_004996	TCONS_00014060	CR45309	FBtr0345583	
	XLOC_011009	TCONS_00031884	CR46216	FBtr0347511	
	XLOC_013478	TCONS_00039592	CR45966	FBtr0347079	
	XLOC_015885	TCONS_00046343	CR45651	FBtr0346368	
	XLOC_017217	TCONS_00050718	CR46016	FBtr0347170	
	XLOC_018845	TCONS_00055658	CR45573	FBtr0346231	
	XLOC_023269	TCONS_00062804	CR45519	FBtr0346055	
